# Early detection and population dynamics of *Listeria monocytogenes* in naturally contaminated drains from a meat processing plant

**DOI:** 10.3389/fmicb.2025.1541481

**Published:** 2025-04-09

**Authors:** Annette Fagerlund, Trond Møretrø, Merete Rusås Jensen, Solveig Langsrud, Birgitte Moen

**Affiliations:** Department of Food Safety and Quality, Nofima – Norwegian Institute of Food, Fisheries and Aquaculture Research, Ås, Norway

**Keywords:** persistence, quasimetagenomics, metagenomics, microbiome, microbiota, subtyping, strain-level typing, food processing environment

## Abstract

*Listeria monocytogenes*, a significant foodborne pathogen, often contaminates ready-to-eat foods through cross-contamination in food processing environments, and floor drains represent one of the most common sites of persistence. Subtyping of *L. monocytogenes* from food processing plants for the purpose of source tracking is usually performed on a single colony obtained after selective enrichment. This study investigates the temporal variation and population dynamics of *L. monocytogenes* in drains, focusing on the diversity of *L. monocytogenes* and the impact of the resident microbiota. Six different drains in a meat processing plant were each sampled four times over a period of 8 weeks and subjected to two-step selective enrichment in Half Fraser and Full Fraser broths. The clonal complexes (CCs) of at least 20 individual *L. monocytogenes* isolates from each positive sample (460 isolates in total) were determined using either the Geno*Listeria* Multiplex qPCR assay or whole genome sequencing (WGS). The microbiota in drains and enrichment cultures was analyzed by 16S rRNA gene amplicon sequencing and metagenomic or quasimetagenomic sequencing. *L. monocytogenes* was detected in the majority of samples and four different CCs were identified – CC9, CC11 (ST451), CC121 and CC8 – with up to three CCs in the same sample and with different CCs dominating in different drains. The same clones of CC9, CC11, and CC121 had persisted in the facility for 3–5 years. The composition of the drain microbiota remained relatively stable over time, with *Pseudomonas*, *Acinetobacter*, *Janthinobacterium*, *Chryseobacterium*, *Staphylococcus*, and *Sphingomonas* as the most commonly identified genera. There were no apparent differences in the microbial genera present in *L. monocytogenes* positive and negative drains or samples. The study highlights the use of techniques such as qPCR and quasimetagenomics for monitoring and controlling the risk of *L. monocytogenes* contamination in processing environments.

## 1 Introduction

*Listeria monocytogenes*, the causative agent of listeriosis, is one of the most concerning foodborne human bacterial pathogens. Although this pathogen is widely found in the environment, major incidences of listeriosis are caused by consumption of contaminated ready-to-eat (RTE) food. Contamination of the final product is usually due to cross-contamination from the food processing environment (Vaìzquez-Boland et al., 2001; Jordan et al., 2018). It is well-known that *L. monocytogenes* can colonize niches such as equipment, floors, and drains in a food processing plant over extended time-periods (a situation known as persistence), and lead to recurring contamination of raw materials and products (Møretrø and Langsrud, 2004; Ferreira et al., 2014). Drains are the most common sites of persistence of *L. monocytogenes* in meat processing plants (EFSA Panel on Biological Hazards (BIOHAZ) et al., 2024). Effective control of *L. monocytogenes* requires a seek-and-destroy strategy, which involves sampling of raw materials and food processing surfaces to identify targets for corrective actions (Food Safety and Inspection Service [FSIS], 2014; Malley et al., 2015). It is essential to distinguish between sporadic occurrences and persistent contamination of *L. monocytogenes*, as these require different eradication strategies. To detect persistence, the sampling and analysis methods must be capable of identifying *L. monocytogenes*, even at low levels, each time the same area is sampled. This typically involves sampling a large area with sufficient mechanical force to detach biofilm followed by selective enrichment. Ideally, recurrent *L. monocytogenes* should be subtyped to confirm the presence of the same clone over time. Consistent results may be challenging to obtain if a sample point contains multiple clones, as usually only one isolate from each sampling point is subtyped.

The resident microbiota can influence the growth, attachment, and biofilm formation of *L. monocytogenes* (Fox et al., 2014; Fagerlund et al., 2021). A proactive control strategy would be to identify and eradicate bacterial communities that promote *L. monocytogenes* attachment and biofilm production. Potential indicator bacterial taxa associated with higher abundance or detection of *L. monocytogenes* have recently been identified from fruit packing facilities (*Pseudomonas*, *Stenotrophomonas*, and *Microbacterium*; Rolon et al., 2023), from a frozen vegetable processing facility (*Enterobacter*, *Serratia*, and *Carnobacterium*; Pracser et al., 2024), from meat samples from a meat processing facility (*Pseudomonas*, *Acinetobacter*, and *Janthinobacterium*; Zwirzitz et al., 2021), and from fabrication and processing rooms of a small meat processing facility (*Acinetobacter*, *Chryseobacterium*, and *Psychrobacter*; Belk et al., 2022). Other studies have identified bacterial taxa that were negatively associated with *L. monocytogenes* occurrence or growth, e.g., from meat conveyor surfaces in a swine slaughterhouse (*Herminiimonas*, *Bryobacter*, *Caulobacter*, *Sphingomonas*, and *Mycobacterium*; Cherifi et al., 2022) and from mushroom processing environments (*Enterobacteriaceae* and *Lactococcus*; Lake et al., 2023). Other studies report no detected associations in this regard (Diaz et al., 2025), and so far, there does not seem to be a clear consensus on which taxa are associated with the presence or absence of *L. monocytogenes* in food industry environments.

It is well-known that certain genotypes of *L. monocytogenes* are more commonly associated with food processing environments and that different subtypes may have different levels of virulence and consequently food safety risk (Maury et al., 2016; Food and Agriculture Organization [FAO] and World Health Organization [WHO], 2022). Subtyping is therefore essential for source tracking, implementation of effective control measures, and risk-based food safety management in the food industry. The currently employed protocols for detection of *L. monocytogenes* involve a two-step selective enrichment and subsequent confirmatory biochemical or molecular species identification (International Organization of Standardization [ISO], 2017). This step may then be followed by genetic subtyping, commonly of one single isolate per positive sample. The length of time from sampling to *L. monocytogenes* detection and identification of subtype is a huge barrier for timely implementation of proper corrective actions, e.g., identification and removal of reservoirs in food processing environments or contaminated products on the market. Thus, there is a need for rapid methods for both detection and subtyping of *L. monocytogenes*.

Prior to the introduction of MLST in 1998 (Maiden et al., 1998), bacteria were typically subtyped using classical immunological serotyping, which distinguishes bacteria within a species based on the detection of specific surface structures (O- and H-antigens). *L. monocytogenes* serotyping, developed in the late 1970s (Seeliger and Höhne, 1979), differentiated *L. monocytogenes* into 13 serotypes, four of which are common among isolates from food and clinical cases (1/2a, 1/2b, 1/2c, and 4b). After the first *L. monocytogenes* whole genome sequences became available in the early 2000’s, a DNA-based molecular analysis, based on PCR amplification of six genes, was developed as a simpler method for distinguishing between different serotypes (Doumith et al., 2004; Kérouanton et al., 2010; Vitullo et al., 2013). The assay could differentiate five PCR-serogroups based on a pattern of presence or absence of specific marker genes or gene variants. For example, the gene *lmo1118* was found to only be present in *L. monocytogenes* of PCR-serogroup IIc (serotypes 1/2c and 3c).

The phylogenetic structure of the species *L. monocytogenes* is now known to consist of well-defined, tight clusters of closely related strains, which largely correspond to clonal clusters (CCs) defined by the classical seven-gene MLST (Chen et al., 2016; Moura et al., 2016). *L. monocytogenes* MLST sequence types (STs) are defined as the unique association of alleles from seven housekeeping genes, and a CC is formed by grouping STs that share alleles at six out of the seven loci (Ragon et al., 2008). Most recent studies employ whole genome sequencing (WGS) for determination of MLST CCs, as it has become cost-effective relative to the classical method employing PCR followed by Sanger sequencing. Furthermore, it simultaneously provides data enabling subsequent higher resolution comparison of isolates within the same CC, which is essential for determining whether two or more isolates are sufficiently related to indicate a common source (Pightling et al., 2018). However, both sequencing-based approaches are relatively time-consuming and expensive compared with PCR-based analyses, limiting their potential for analysis of larger datasets and routine surveillance in the food industry.

To address the need for faster and more convenient subtyping, several new analysis methods have recently been developed based on the same principle as PCR-serogrouping, but with more marker genes (ranging from 13 to 31). These are more detailed attempts to correlate patterns of presence or absence of genetic markers with prevalent genetic populations of *L. monocytogenes* (i.e., CCs). Examples include GENE-UP Typer from bioMérieux^1^, the Listeria PatternAlert assay from Rheonix^2^, and the GenoListeria Multiplex assay developed by ANSES, which distinguishes between 30 CCs widely circulating in Europe (Félix, 2023; Félix et al., 2023). Although these approaches are faster than traditional MLST and WGS, they are similarly affected by the potential loss of diversity when selecting a single colony for analysis, as different subtypes of *L. monocytogenes* may be present in the same sample (Döpfer et al., 2008; Chen et al., 2020; Stessl et al., 2020; Wagner et al., 2021; Acciari et al., 2022).

Another potential reason for failure to detect all *L. monocytogenes* variants that may contaminate the product is enrichment bias, resulting in a change in the relative proportion of *L. monocytogenes* subtypes during the course of the selective enrichment. Previous work by our group and others have shown that *L. monocytogenes* subtypes have slightly different growth potential in Fraser broth (Wagner et al., 2021; Rosa Rodrigues de Souza et al., 2023). However, when *L. monocytogenes* strains were co-enriched with a mock community background microbiota composed of strains capable of growing in Fraser broth, the relative proportions of the different *L. monocytogenes* STs remained relatively consistent during selective enrichment (Wagner et al., 2021). In that study, we also demonstrated that quasimetagenomic sequencing (metagenomic shotgun sequencing of enrichment cultures; Ottesen et al., 2020) could be used for faster identification of *L. monocytogenes*. Additionally, Illumina sequencing enabled the prediction of the presence of co-occurring *L. monocytogenes* strains. Although methods exist for mapping of sequence read data to MLST databases (Zolfo et al., 2017; Clausen et al., 2018), extracting strain-level subtyping information from metagenomic data remains challenging, even though several software tools for strain-level identification from metagenomic data have been developed in recent years (Anyansi et al., 2020; Ghazi et al., 2022; Liao et al., 2023; Lindner et al., 2024).

In this study, the Geno*Listeria* Multiplex qPCR assay, WGS, 16S rRNA gene amplicon sequencing and metagenomic or quasimetagenomic sequencing was employed to examine the temporal variation and population dynamics of *L. monocytogenes* CCs and the co-occurring residential microbiota in naturally contaminated drains in a meat processing plant. A second objective was to assess the impact of the resident microbiota on *L. monocytogenes* presence in drains and during selective enrichment.

## 2 Materials and methods

### 2.1 Sampling and culture enrichment

Samples from six drains in a meat processing facility were collected using sampling cloths (swab cloths 32 × 40 cm, with 25 mL buffered peptone water with 10% neutralizing solution; SodiBox; cat no. 3040) on four occasions over a total period of 9 weeks. During the sampling period the facility, including the drains, was subjected to the cleaning and disinfection procedures routinely in use in the facility. After sampling, the cloths were stored at 4°C and analyzed within 2 h using the ISO 11290-1 method (International Organization of Standardization [ISO], 2017). Bags with sample cloths were added 100 mL Fraser broth base (Oxoid) and stomached for 1 min before the initial samples were collected (samples taken prior to enrichment). Half Fraser selective supplement (Oxoid) was then added to the remaining sample, before primary enrichment at 30°C for 24 h. Then, 100 μL culture was transferred to 10 mL Full Fraser broth (Oxoid) for secondary enrichment at 37°C for 24 h. Samples were withdrawn before start of enrichment, after 4 and 24 h enrichment in Half Fraser broth and after 24 h enrichment in Full Fraser broth. To determine cell counts, dilutions of the enrichment cultures were plated on *Listeria*-selective RAPID *L. mono* agar (RLM; Bio-Rad) and Standard Plate Count Agar (PCA; Oxoid). RLM plates were incubated at 37°C for 1–2 days, while the PCA plates were incubated for 3–5 days at 20°C. Plating on RLM was not performed for the drain samples prior to enrichment in the first week of sampling. For metagenomic analysis, samples were collected by withdrawing 4 mL culture (at the start and after 4 h) or 1 mL culture (after 24 h in Half and Full Fraser), centrifugation at 13,000 *g* for 5 min, washing once in 500 μL TE buffer, and then storage at –20°C.

Statistical differences for TVCs obtained from each swab cloth (prior to enrichment) was assessed using Minitab v.22 software and two-way analysis of variance (ANOVA) with drains and weeks as factors. TVCs were log transformed prior to statistical analysis to stabilize variance and normalize the data. The residuals met the assumptions of normality, homogeneity of variances, and independence. The tested null hypotheses were that there were no differences in TVCs between drains or weeks. After rejecting the null hypotheses (*p* < 0.05), Tukey’s *post-hoc* test for pairwise comparisons was performed to compare differences between factors.

### 2.2 DNA extraction

For isolation of DNA from single isolates, single colonies were picked from RLM agar plates, inoculated in 5 mL Brain heart infusion (BHI) broth (Oxoid), and grown overnight at 37°C with shaking. The pellet from 800 μL culture was suspended in 500 μL of 2x Tris-EDTA buffer with 1.2% Triton X-100. Cells were lysed using lysing matrix B and a FastPrep instrument (both from MP Biomedicals), and genomic DNA was isolated using the DNeasy blood and tissue kit (Qiagen).

Isolation of DNA from samples from enrichment cultures was performed using DNeasy PowerLyzer Powersoil (Qiagen) according to the manufacturer’s instructions; cells were lysed using a Precellys Evolution Instrument (Bertin Technologies) at 7,400 rpm for three rounds of 40 s.

### 2.3 Real-time qPCR for MLST subtyping

The Geno*Listeria* multiplex qPCR method (Félix, 2023; Félix et al., 2023) was performed as described with some modifications. Simplex PCR reactions were performed using primers and probes for CC9 (PCR-serogroup IIc), CC11-ST451, CC121, CC199, and CC14-ST91-ST160-ST360. Isolates with no positive amplification in the simplex PCR reactions were analyzed using a *prs* and *plcA* multiplex qPCR to identify isolates as *Listeria spp.* and *L*. *monocytogenes*. Amplifications with Ct < 18 were considered positive. The remaining isolates were identified using WGS.

All probes were labeled with FAM/BHQ-1 except the *plcA* probe, which was labeled with VIC/BHQ-1. qPCR reactions were run in duplicates with 10 μL reaction volumes each containing 1 μL DNA, 5 μL 2x PerfeCTa qPCR ToughMix low ROX (Quantabio), and 0.3 μM each of probe, forward primer, and reverse primer. DNA concentrations were in the range 5–15 ng/μL. Amplification conditions with Fast Program on QuantStudio5 (BioTek) were: 95°C for 2 min, 40 cycles of 95°C 3 s and 60°C for 30 s.

### 2.4 WGS and genome assembly

Libraries for WGS were prepared using the Nextera XT DNA sample preparation kit (Illumina) and sequenced on a MiSeq platform with 300 bp paired-end reads. Raw reads were filtered on q15 and trimmed of adaptors before *de novo* genome assembly was performed using SPAdes v.3.13.0 (Bankevich et al., 2012) with the careful option and six *k*-mer sizes (21, 33, 55, 77, 99, and 127). Contigs with sizes of < 500 bp and *k*-mer coverage of < 5 were removed from the assemblies. The average coverage for the genome assemblies was calculated using BBmap v36.92 (Bushnell, 2014) and the quality of all assemblies was evaluated using QUAST v5.0.2 (Mikheenko et al., 2018). The assemblies were annotated using the NCBI Prokaryotic Genomes Annotation Pipeline (PGAP) server.

A total of 97 *L. monocytogenes* isolates were collected from the factory during 2017–2019 as part of an earlier study (Fagerlund et al., 2022), and of these, 38 were previously subjected to WGS [National Center for Biotechnology Information (NCBI) BioProject accession PRJNA689484 and European Nucleotide Archive (ENA) Project PRJEB56155]. For the 14 isolates in PRJEB56155 (Ivanova et al., 2025), raw sequencing data was subjected to genome assembly as described above. The genomes analyzed in the current study are listed in Supplementary Table 1. Basic Local Alignment Search Tool (BLAST) analyses were performed using blastn v2.12.0+ integrated in CLC Main Workbench 22.0.2 (Qiagen). Alignments were generated using CLC Main Workbench.

### 2.5 16S rRNA gene amplicon sequencing

16S rRNA gene PCR (V4 region) and paired-end sequencing (2 × 150 bp) using the MiSeq reagent kit v3 on a MiSeq instrument (Illumina) were performed following the protocol by Caporaso et al. (2012) as previously described (Møretrø et al., 2021). The sequences were processed in QIIME 2 (qiime2-2023.5) (Bolyen et al., 2019). Briefly, raw data were demultiplexed using the q2-demux plugin, followed by joining paired ends with vsearch (Rognes et al., 2016). Quality filtering was based on a q-score above 30, and denoising was performed using deblur-16S (Amir et al., 2017). Taxonomy was assigned to sOTUs using the q2-feature-classifier (Bokulich et al., 2018) classify-sklearn naiïve Bayes taxonomy classifier against the SILVA 16S database (Silva 138 99% OTUs from the 515F/806R region) (Quast et al., 2013). Singletons were removed, and mitochondrial and chloroplast sequences were filtered out before collapsing the taxonomy tables to genus identity level (L6), converting to relative values, exporting to text files, and further processing in Microsoft Excel. All sOTUs were aligned with mafft (Katoh and Standley, 2013) via q2-alignment and used to construct a phylogeny with fasttree2 (Price et al., 2010) via q2-phylogeny. Alpha and beta diversities were estimated using q2-diversity (core-metrics-phylogenetic) (Bolyen et al., 2019) with a sampling depth of 26,400 sequences. The results for the different metrics were visually inspected, and the results from the Shannon and Bray-Curtis metrics were used for calculation and visualization in Principal Coordinate Analysis (PCoA) plots. Differences in Shannon index were calculated using ANOVA (GLM and one-way) in Minitab v21.4.3, and the bacterial community diversity (Bray-Curtis distance) across different parameters was statistically evaluated using pairwise permutational multivariate analysis of variance (PERMANOVA) tests in QIIME 2 (Anderson, 2001; Anderson and Walsh, 2013).

### 2.6 Quasimetagenomic shotgun sequencing

For the selected samples from the *L. monocytogenes* culture enrichments, 200 ng of genomic DNA was subjected to paired-end sequencing (2 × 301 bp). Briefly, libraries were prepared as described in the Illumina DNA prep reference guide (Illumina DNA prep kit; Nextera DNA CD indexes; Illumina). Samples were purified, quantified with Qubit HS dsDNA (Invitrogen), normalized, and pooled. The sample pool was purified, quantified, and diluted to 4 nM prior to a denaturation and dilution procedure provided by Illumina, including the use of 8 pM DNA input og 5% PhiX spike.

Taxonomic classification was performed as previously described (Wagner et al., 2021). Illumina reads were filtered on q15 and trimmed of adaptors using fastq-mcf from the ea-utils package (Aronesty, 2011). Taxonomic classification of the filtered Illumina reads was performed using the *k*-mer approach employed in Kraken2 v2.1.1 (Wood et al., 2019) and the available pre-built Kraken2 database PlusPFP (containing indexes for the archaea, bacteria, viral, plasmid, protozoa, fungi, plant, human, and UniVec_Coreplus RefSeq databases from 27 January 2021). A confidence score threshold of 0.05 was selected, and the minimum base quality used in classification was 30.

### 2.7 MLST and strain-level subtyping

Classical MLST analysis followed the MLST scheme described by Ragon et al. (2008) and the database maintained at the Institute Pasteur’s *L. monocytogenes* online MLST repository^3^. The wgMLST analysis was performed using a whole-genome scheme containing 4,797 coding loci from the *L. monocytogenes* pan-genome and the assembly-based BLAST approach, implemented in BioNumerics 8. The minimum spanning tree was constructed using BioNumerics based on the categorical differences in the allelic wgMLST profiles for each isolate. Loci with no allele calls were not considered in the pairwise comparison between two genomes. The number of allelic differences between isolates was read from genetic distance matrices computed from the absolute number of categorical differences between genomes.

Mapping of Illumina reads to the Institute Pasteur’s *L. monocytogenes* MLST database (Ragon et al., 2008) was performed as previously described (Wagner et al., 2021). Illumina reads classified to Taxon IDs 1637 (*Listeria* spp.) and 1639 and below (*L. monocytogenes* classified to species and strain level) using Kraken2 were extracted to file using the KrakenTools (Lu et al., 2022) and mapped to the Institute Pasteur’s *L. monocytogenes* MLST database using the KMA mapping program (Clausen et al., 2018).

MetaMLST v1.2.1 (Zolfo et al., 2017) was used to detect the dominant ST in metagenomic shotgun sequencing data. A MetaMLST database was built for *L. monocytogenes* using metamlst-index.py, from loci sequence files and MLST profiles downloaded from the Institute Pasteur’s *L. monocytogenes* MLST repository on 14.01.2021. Illumina reads were mapped to the MetaMLST database using bowtie2 v2.3.5.1 (Langmead and Salzberg, 2012) and parameters --very-sensitive-local -a --no-unal. MLST loci contained in the sample were detected using metamlst.py and STs were called using metamlst-merge.py.

StrainScan v1.0.14 was used to perform strain-level composition analysis of the metagenomic shotgun sequencing data. Two reference databases were used, one built with one representative genome from each of six CCs; CC9 (MF8690; contains *lmo1118*), CC11-ST451 (MF8691), CC121 (MF8693), CC8 (MF8681), CC199 (MF7408), and CC14-ST91 (MF7327), and the other with all genomes listed in Supplementary Table 1. StrainScan was run using both databases and both with default settings and with the parameter --low_dep 2.

## 3 Results

### 3.1 Sampling in the meat processing facility

**FIGURE 1:**
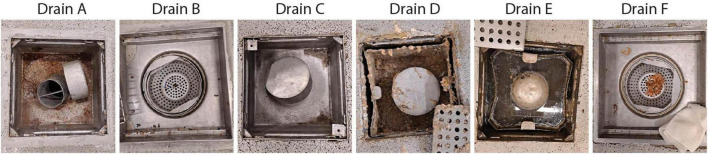
Representative pictures of the six sampled drains. The swab cloth used for sampling is shown next to drain F.

Six drains from a high risk zone in a meat processing facility, referred to as drains A to F (Figure 1), were selected for sampling. Drains A, B, and F were dry or relatively dry, and located adjacent to each other in the same region of the processing department. They received relatively limited amounts of soiling and effluents from the meat processing operation. Drains C, D, and E were usually humid when sampled, and located in another area of the same department. Drain D, located in a cooling room, had the highest load of soiling and effluent. The sampling was performed in four different weeks in 2022, each time early during the first work shift on each sampling day. The first and second sampling time-points were 6 weeks apart, while the three last sampling occasions occurred in consecutive weeks. These time-points are referred to as weeks 1, 7, 8, and 9. All drains were cleaned with a chlorinated alkaline foam-based cleaning agent, and standard cleaning routines were followed throughout the sampling period.

### 3.2 *L. monocytogenes* was detected in the majority of drains

**FIGURE 2:**
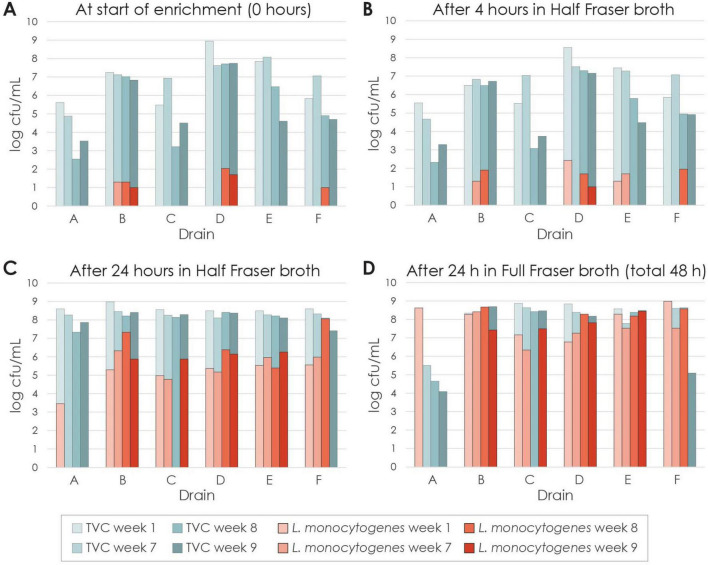
Bacterial concentrations measured as total viable counts (TVC) and counts of *L. monocytogenes*. **(A)** Samples from swab sampling cloths after suspension in 100 mL volume, before the start of enrichment. After **(B)** 4 h and **(C)** 24 h of primary enrichment in Half Fraser broth, and **(D)** 24 h secondary enrichment in Full Fraser broth. Samples were not analyzed for *L. monocytogenes* before start of enrichment in week 1. The detection limit was 10 cfu/mL and results below this limit are not indicated. Culture volumes were 100 mL for Half Fraser and 10 mL for Full Fraser enrichments.

The swab cloths were subjected to two-step selective enrichment for *L. monocytogenes*, including a 24 h primary enrichment step in Half Fraser broth followed by 24 h secondary enrichment in Full Fraser broth (International Organization of Standardization [ISO], 2017). The cultures were analyzed for both total bacterial counts (total viable counts; TVC) and counts of *L. monocytogenes* at four time-points: before enrichment, after 4 and 24 h primary enrichment in Half Fraser broth (t4 and t24), and after secondary enrichment; 24 h after subculturing into Full Fraser broth (t48) (Figure 2). Of the 24 analyzed samples, only five were negative for *L. monocytogenes*. Drain A was only positive for *L. monocytogenes* during the first sampling week and drains C and F were negative for *L. monocytogenes* in weeks 8 and 9, respectively. The remaining three drains were positive for *L. monocytogenes* in all four sampling weeks.

All 19 samples that were *L. monocytogenes* positive after secondary enrichment in Full Fraser broth were also positive after 24 h primary enrichment in Half Fraser broth. However, the proportion of *L. monocytogenes* relative to the total bacterial counts in the positive cultures increased in the second step, from median values of 0.3% in Half Fraser (t24) to 55% in Full Fraser broth (t48).

Of the 18 samples collected during weeks 7–9, 13 were positive for *L. monocytogenes*, and for six of these, presumptive *L. monocytogenes* were detected prior to enrichment. The detection limit was 10^3^ cfu per sample (International Organization of Standardization [ISO], 2017). As 1–11 colonies were obtained on the selective agar plates, samples from the drains with the highest concentrations appeared to contain around 10^3^–10^4^ *L. monocytogenes* bacteria. All six samples from week 1, which were not analyzed for *L. monocytogenes* prior to enrichment, tested positive, with two samples showing detection at t4.

A color change from yellow to dark brown in Fraser broth is due to hydrolysis of esculin, and is a presumptive indication of the presence of *L. monocytogenes* and other *Listeria* spp. All the *L. monocytogenes* positive cultures showed this color change after primary enrichment in Half Fraser broth. In addition, one of the five samples that were negative for *L. monocytogenes* – drain C in week 8 – showed the same color change during secondary enrichment in Full Fraser broth, but not in Half Fraser. The other four *L. monocytogenes* negative samples had, on average, 2.9 log lower TVCs (ranging from 2.3 to 3.8 log lower) after 24 h secondary enrichment in Full Fraser (t48) compared to 24 h primary enrichment in Half Fraser broth (t24). In contrast, the sample from drain C in week 8 did not show a lower TVC in Full Fraser compared to in Half Fraser. Thus, for the *L. monocytogenes* negative samples, the presence of bacteria capable of hydrolysing esculin appeared to correlate with the presence of bacteria that could grow well in Full Fraser broth.

### 3.3 Diversity of total microbiota in the drains

The total counts of bacteria in the drain samples were relatively high overall, with on average 8.1 log TVC (SE ± 0.4) obtained from each swab cloth (prior to enrichment). The variation was relatively large, from 4.5 log to 11.0 log TVC per drain sample (i.e., per total swab cloth). The main effects of both drains and weeks were statistically significant (two-way ANOVA, *p* < 0.005), and the TVCs were significantly lower in drains when sampled in weeks 8 and 9 relative to in weeks 1 and 7 (Tukey’s *post-hoc* test, *p* < 0.04). It should be emphasized that no special cleaning measures were implemented during the sampling period, so the explanation behind this reduction in TVC is not known.

The microbiota in the drains and enrichment cultures was studied using Illumina sequencing of 16S rRNA gene amplicons, a technique also known as metabarcoding. Nine samples, including the samples taken prior to enrichment in drains A, B, and C in week 8, failed to generate sufficient DNA for sequencing after PCR due to too low DNA concentration. In total, 87 samples were analyzed, resulting in 6.9 million sequences after denoising and quality filtering. This yielded 1,738 bacterial sequences down to single nucleotide differences (suboperational taxonomic units; sOTUs). Identification to the genus level revealed 527 taxa, of which 18 had an average relative abundance of > 1% across all samples or a maximum average abundance of > 10% in any single sample.

**FIGURE 3:**
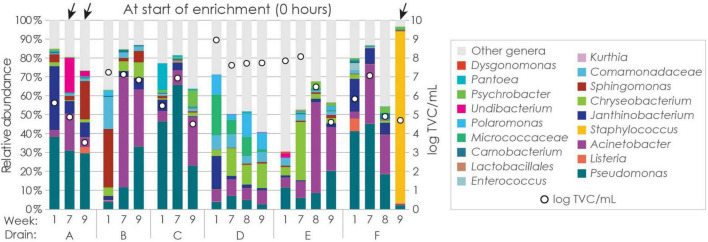
Relative abundance (%) of bacterial genera in the drain samples based on 16S rRNA gene amplicon sequencing. Taxa with abundance above 1% across all samples and/or at least 10% abundance in any one sample (considering all time-points) are presented, and remaining taxa are presented in the “other genera” category. Samples negative for *L. monocytogenes* are indicated by arrows. Bacterial concentrations on the secondary axis are for swab sampling cloths after suspension in 100 mL volume.

The most commonly identified genus in the drains (prior to enrichment) was *Pseudomonas*, with average and median relative abundances of 22% and 19%, respectively, across the 21 analyzed samples (Figure 3). Other frequently identified genera included *Acinetobacter* (15%), *Janthinobacterium* (6%), and *Chryseobacterium* (5%). *Staphylococcus* dominated with 91% relative abundance in one sample (drain F in week 9), but the median abundance across samples was only 0.05%. *Sphingomonas* was highly prevalent in two samples – drain A in week 9 and drain B in week 1 – with relative abundances of 20% and 31%, respectively. However, the overall median relative abundance of *Sphingomonas* was relatively low at 0.8%.

*Listeria* constituted only a minority of the total drain microbiota, with average and median relative abundances of 0.6% and 0.1%, respectively. Only two drains had relative abundances of *Listeria* above 0.2%: drain A in week 9 (2.3%) and drain F in week 1 (6.7 %). Notably, drain A in week 9 tested negative for *L. monocytogenes*, however, only one sOTU was identified for the *Listeria* genus, meaning that the analysis cannot distinguish *Listeria* below genus level. Presumably, since *L. monocytogenes* was not detected, the 16S rRNA gene sequencing reads from drain A in week 9 represent *Listeria* spp. other than *L. monocytogenes*.

Due to the inherent limitations of 16S rRNA gene amplicon sequencing, the detection and accurate quantification of low abundance bacteria can be challenging, often leading to increased uncertainty in the results for rare taxa (Bonk et al., 2018). Nevertheless, considering the TVCs and relative abundances of *Listeria* in each sample, the number of *Listeria* cells per drain sample was calculated to range from 10^4^ to 10^8^ cfu, with a median of 7 × 10^5^ cfu. These estimates for *Listeria* spp. were orders of magnitude higher than those estimated for *L. monocytogenes* from direct plating of samples from weeks 7 to 9 on selective agar plates (≤10^4^ cfu per drain sample).

Sequencing data was obtained prior to enrichment for three of the five drain samples in which *L. monocytogenes* was not detected. Drain A was positive for *L. monocytogenes* in week 1, and negative the other weeks, however, no apparent difference in the microbiota was observed between these weeks (Figure 3). For drain F, however, there was a significant shift in the microbiota in week 9, when no *L. monocytogenes* was detected and the drain was completely dominated by *Staphylococcus*, compared to in weeks 1, 7, and 8, when the drain was positive for *L. monocytogenes* and the microbiota was more similar to that of the other analyzed samples.

**FIGURE 4:**
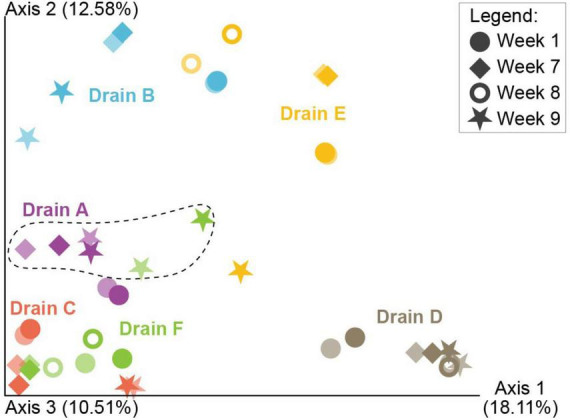
Principal Coordinates Analysis (PCoA) based on Bray Curtis dissimilarity depicting taxonomic differences among samples taken before enrichment (represented by weakly colored icons) and after 4 h of primary enrichment (represented by strongly colored icons), analyzed using 16S rRNA gene amplicon sequencing. Distinct colors indicate different drains, while different symbols indicate different weeks. Samples circled by the dashed line were from drains that tested negative for *L. monocytogenes* after selective enrichment.

The microbiota in the drains prior to enrichment was highly similar to that observed after 4 h of primary enrichment (Figure 4 and Supplementary Figure 1A). No significant difference was seen between samples collected at these two time-points in Shannon diversity (microbial richness, i.e., the number of taxonomic groups, and evenness, i.e., distribution of abundances; alpha diversity, *p* = 0.979) or in Bray-Curtis distances (microbial diversity; beta diversity, *p* = 0.999). This is also evident from the PCoA plot shown in Figure 4, although the samples from drain D (cooling room) diverge somewhat from the samples from the other drains.

For evaluation of the influence of the microbiota on the prevalence of *L. monocytogenes*, samples collected both directly from the drains and after 4 h of enrichment were included (*n* = 41), in order to increase the number of samples for analysis. There was a significant difference in the Shannon diversity between the different drains (*p* = 0.017), where drains B and F (dry or relatively dry) had significantly lower diversity than drains D and E (usually humid) (*p* < 0.001). There was also a significant difference in Bray-Curtis distances between drains (*p* = 0.001, test statistic = 5.19). PERMANOVA analysis also revealed a significant difference in the Bray-Curtis distances between *L. monocytogenes* positive and negative samples (*p* = 0.003, test statistic = 2.46). However, this result was influenced by the low number of *L. monocytogenes* negative drain samples in the dataset and the fact that there was a significant difference between the drains.

The diversity in the total microbiota for the remaining samples collected during selective enrichment is shown in Supplementary Figure 1 and further discussed below, along with data on *L. monocytogenes* population dynamics during enrichment.

### 3.4 qPCR and WGS identified four different CCs

All but one of the presumptive *L. monocytogenes* colonies obtained before enrichment (*n* = 22), all colonies obtained after 4 h of primary enrichment (*n* = 59), and 10 colonies each from positive samples after 24 h enrichment in Half and Full Fraser were isolated for multilocus sequence typing (MLST) identification. In total 460 isolates were collected.

The “Geno*Listeria*” real-time qPCR Taqman subtyping method developed by Félix et al. (2023) was used for MLST subtyping. Initially, qPCR reactions were chosen for the analysis based on which CCs were previously determined to be present in the factory during 2017–2019 (Fagerlund et al., 2022; Ivanova et al., 2025; Supplementary Table 1). In accordance with this, the qPCRs to detect the molecular markers identifying CC9 (PCR-serogroup IIc), CC11-ST451, CC121, CC199, and CC14-ST91-ST160-ST360 (hereafter referred to as CC14-ST91) were sequentially performed for the 460 isolates. According to the published protocol, amplifications should have a cycle threshold (Ct) of less than or equal to 25 cycles (Ct ≤ 25) to be considered positive (Félix, 2023; Félix et al., 2023).

Using this approach, 186 isolates (40%) were identified as CC9 (Ct < 17.4, the remaining 274 had Ct > 25.5), 217 isolates (47%) as CC11-ST451 (Ct < 16.6, the remaining 57 had Ct > 27.5), and 26 isolates (6%) as CC121 (Ct < 14.6). In the CC121 qPCR reactions, two samples obtained Ct ≤ 25 (21.7 and 22.6), but were considered inconclusive in the current study. The remaining 31 isolates were negative also in the CC199 qPCR reaction and inconclusive in the CC14-ST91 qPCR reaction (15 isolates had Ct values in the range 21–25). One of the 31 isolates was then identified as a *Listeria* spp. other than *L. monocytogenes* by qPCR targeting *prs* and *plcA*. In total using this approach, 914 qPCR reactions were run.

The remaining 30 non-identified isolates were typed using WGS. Three isolates belonged to CC8 (0.7%) and 26 isolates (including the two that were inconclusive in the CC121 qPCR) belonged to CC9, bringing the total number of CC9 isolates to 212 (46%). The final isolate was determined to be *Enterococcus faecalis*. The intermediate Ct values observed in the CC14-ST91 qPCR could be attributed to a perfect match in the primer pair and only one mismatch in the TaqMan probe sequence, relative to the genomes of CC8 and CC9.

BLAST analysis showed that the 26 CC9 isolates for which the CC9 qPCR was negative (comprising 12% of all typed CC9 isolates) lacked the genetic marker *lmo1118* used to detect CC9 in the Geno*Listeria* scheme (Félix, 2023; Félix et al., 2023). A total of 22 CC9 isolates collected from the meat processing factory during 2017–2019 were previously typed using WGS (Fagerlund et al., 2022; Ivanova et al., 2025; Supplementary Table 1). Of these isolates, 32% (*n* = 7) lacked *lmo1118*. CC9 isolates lacking *lmo1118* were found in drains B, D, and E. No CC9 isolates from drains A, C, and F were typed using WGS.

The CC9 genome assemblies were fragmented in the region where *lmo1118* is located (when present). Therefore, the region from *lmo1116* to *lmo1122* in the EGD-e genome was aligned with the corresponding regions in the genomes of six completely sequenced CC9 isolates from Norwegian meat processing facilities; MF4626, MF4624, MF4697, MF6172, MF4562, MF4545 (Fagerlund et al., 2018; Supplementary Figure 2). Four of these genomes were identical to EGD-e in this region, while in MF4562, the insertion sequence IS*1542* encoding a transposase (Darini et al., 1999) was inserted on either side of the *lmo1119* and *lmo1118* genes. In MF4545, this cassette was replaced by a third copy of IS*1542*, flanked on either side by an ORF identical to the first half of *lmo2365*.

### 3.5 Persistence and diversity of *L. monocytogenes*

Whole genome MLST (wgMLST) analysis was performed to examine whether the sequenced isolates collected in 2022 were similar across drains, and/or similar to the 38 previously sequenced isolates collected during 2017 to 2019. In addition to the 29 isolates sequenced as a response to negative or ambiguous qPCR results in the current study (CC8 and CC9), five additional randomly selected isolates (identified using qPCR), were also subjected to WGS (and wgMLST) – two CC9, two ST451, and one CC121 (see Supplementary Table 1).

**FIGURE 5:**
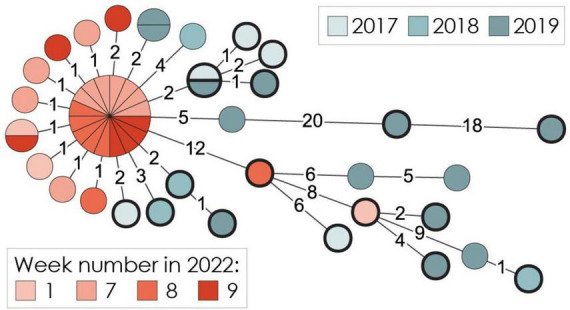
Minimum spanning tree for 50 *L. monocytoge*nes CC9 genomes from the meat processing factory, based on wgMLST analysis using a scheme of 4,797 loci. The area of each circle is proportional to the number of isolates represented, and the number of allelic differences between isolates is indicated on edges connecting the nodes. Nodes representing isolates containing the *lmo1118* marker gene are highlighted by a thick black ring.

Previous work (Fagerlund et al., 2022) identified CC9 as persisting in the meat processing facility (factory M8) over a period of 2 years. Several of the CC9 isolates from 2017 to 2019 were from drain D (*n* = 4) or other drains in the same department as drains A to F. All isolates (22 from 2017 to 2019 and 28 from 2022) belonged to the same clone (Figure 5). The 50 CC9 isolates were differentiated by a maximum of 42 wgMLST allelic differences, with a median genetic distance of four alleles, showing that the same strain had persisted in the facility for at least 5 years (Figure 5). Isolates containing the *lmo1118* qPCR target gene were not genetically distinct from those lacking *lmo1118*.

The two sequenced CC11-ST451 isolates from 2022, from drain A in week 1 and drain F in week 8, differed by only 1 wgMLST allele. An isolate from a product sample collected in 2019 was identical by wgMLST to the currently sequenced isolate from drain A. Thus, it seemed that CC11-ST451 had persisted in the factory for at least 3 years. The sequenced CC121 isolate from drain C in week 7, 2022, showed 2 to 23 wgMLST allelic differences toward the ten CC121 sequenced isolates collected during 2017–2019, including 2 and 3 wgMLST allelic differences toward two isolates collected from drain D in 2019 and 2018, respectively. This means that CC121 had persisted in the facility for 5 years. The three CC8 isolates identified in 2022, from drain D in week 7 and drain E in week 8, showed 3 to 9 wgMLST allelic differences, indicating that the same strain was present in both drains.

In summary, the same clones of CC9, CC11-ST451, and CC121 appeared to have persisted in the facility for 3–5 years. CC8 was only identified in 2022, while CC199 and CC14-ST91 were only detected during 2017–2019. At least one of the four previously analyzed CC199 isolates were from the same department in the factory as drains A to F sampled in the current study.

The CC9 and CC121 persistent clones both carried premature stop codon mutations in the *inlA* gene, which encodes the virulence factor internalin A [mutation types 12 and 6, respectively (Van Stelten et al., 2010)]. These mutations indicate that these clones were hypovirulent (Food and Agriculture Organization [FAO] and World Health Organization [WHO], 2022). The CC11-ST451 persistent clone carried a full-length *inlA*. None of the isolates carried the *Listeria* pathogenicity islands LIPI-3 or LIPI-4. All three persistent clones carried a *repA*-family theta-replicating plasmid, with sizes of 26 kb, 61 kb and 66 kb in the CC9, CC121, and CC11-ST451 clones, respectively. The plasmids in the CC9 and CC121 clones contained a cadmium resistance operon (*cadA1C1*) and the *clpL* heat resistance determinant. Additionally, both CC9 and CC121 harbored the Tn*6188* transposon encoding QacH, conferring resistance to quaternary ammonium compound (QAC) sanitizers (Müller et al., 2014). The CC9 clone also carried stress survival islet 1 (SSI-1), promoting growth under low pH and high salt stress conditions, while the CC121 clone contained SSI-2, which provides protection against alkaline or oxidative stress (Ryan et al., 2010, Harter et al., 2017). Furthermore, the CC9 clone harbored the Tn*554*-like transposon carrying the arsenic resistance cassette *arsCBADR*. These features suggest that both the CC9 and CC121 clones are classical food-processing-associated clones, well-adapted to such environments. In contrast, the CC11-ST451 clone lacked all queried stress and resistance genes (Fagerlund et al., 2022).

### 3.6 Population dynamics during selective enrichment

**FIGURE 6:**
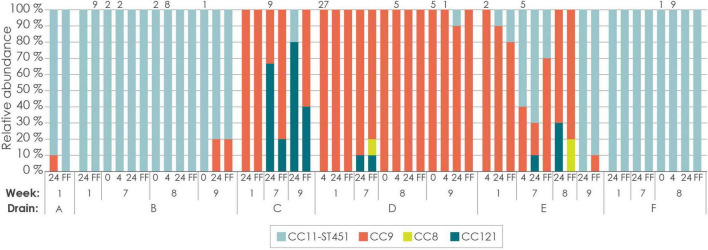
Distribution of *L. monocytogenes* CCs determined by subtyping of single isolates. Each column shows results from identification of 10 isolates, except where otherwise indicated by a number above the column. Labels 0, 4, and 24 refers to time-points before enrichment, 4 and 24 h primary enrichment in Half Fraser broth, respectively. FF indicates isolates sampled after secondary enrichment in Full Fraser broth.

Different CCs dominated different drains (Figure 6). The three driest drains (A, B, and F) located in one end of the production room were almost completely dominated by CC11-ST451, while the predominant type in the three more humid drains located in another area of the room (C, D, and E) was CC9. CC121 was detected sporadically in two drains (D and E) and 2 weeks in a row in one (drain C), and no isolates belonging to CC121 or CC8 were detected during week 1.

As presented above, the microbiota in the drains prior to enrichment was highly similar to that observed after 4 h of primary enrichment (Figures 3, 4 and Supplementary Figure 1A). In the five drains where *L. monocytogenes* colonies were obtained at these two time-points, the same CC was exclusively obtained from each drain, except in the case of drain D in week 9, where one of ten colonies obtained after 4 h enrichment belonged to a different CC (Figure 6). This indicates a stable microbial community composition, consistent with the absence of enrichment bias in the *L. monocytogenes* population. However, it should be noted that the number of *L. monocytogenes* colonies was low at the early sampling times.

After 24 h of primary enrichment, the dominance of *Pseudomonas* had increased to 67% for both average and median relative abundances, and a substantial increase in the phylum *Firmicutes* was observed (Supplementary Figure 1B). After secondary enrichment in Full Fraser broth, the microbiota changed dramatically and became much less diverse compared to in Half Fraser broth (Supplementary Figure 1C). All secondary enrichments for drains that were positive for *L. monocytogenes* were dominated by *Listeria*, except drain B in week 1 which contained 78% *Enterococcus*. These changes coincided with a noticeable trend of higher proportions of CC121 after 24 h primary enrichment in Half Fraser broth, which seemed to be outcompeted by CC9 after secondary enrichment in Full Fraser broth. The opposite tendency was found for CC8, which was only isolated after secondary enrichment. Given the limited data, it would be highly speculative to conclude whether the microbiota in the enrichment cultures contributed to the observed trends in CCs, or if the observed trends were due to enrichment bias favoring certain CCs in Half Fraser and Full Fraser. No clear associations between the microbiota during enrichment and the presence of specific CCs were apparent from the data.

Regarding the microbiota in the secondary enrichment cultures for drains that did not contain *L. monocytogenes*, four of the five samples were dominated by either *Pseudomonas* or *Staphylococcus* (Supplementary Figure 1C). The fifth *L. monocytogenes* negative sample, from drain C in week 8, showed a color change to brown in Full Fraser broth, indicating the presence of bacteria capable of hydrolysing esculin. This sample had a relative abundance of 99% *Listeria* in the secondary enrichment culture, presumably representing reads from *Listeria* spp. other than *L. monocytogenes*.

### 3.7 Diversity at species-level analyzed by shotgun metagenomics

To examine species distribution below genus level, samples from one of the drains which contained more than one *L. monocytogenes* ST – drain E in week 1 – was selected for further characterization. The samples collected prior to enrichment, during primary enrichment (t4 and t24), and after secondary enrichment were analyzed using shotgun metagenomics with Illumina sequencing. This analysis is usually termed “quasimetagenomics” when applied to selective enrichment cultures (Ottesen et al., 2020). The sequencing yielded between 3.6 and 7.2 million paired-end reads per sample. The selected drain sample was determined to be positive for *L. monocytogenes* after 4 h of primary enrichment, and ST9 and CC11-ST451 were detected after both primary and secondary enrichment.

**FIGURE 7:**
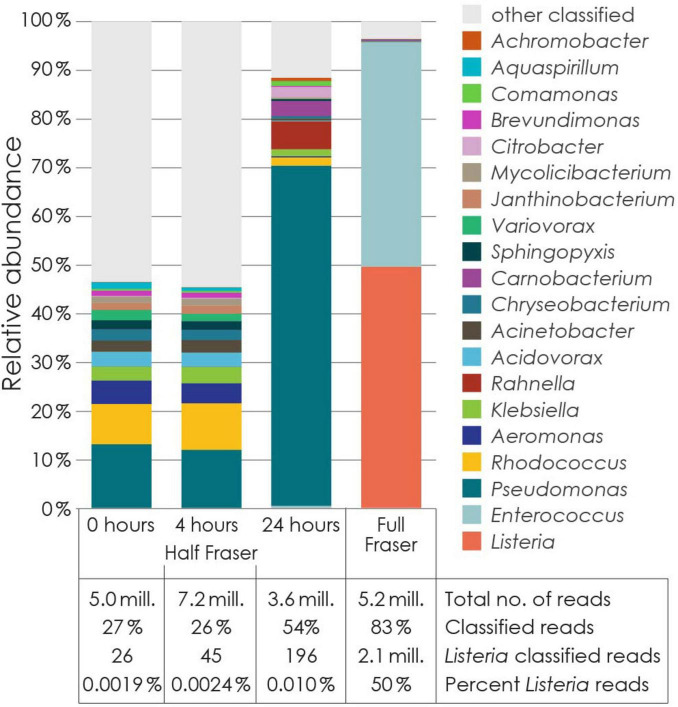
Genus level taxonomic assignment of reads from Illumina MiSeq metagenomic and quasimetagenomic shotgun sequencing of samples from drain E in week 1. Classified reads from genera with< 0.3% abundance (of total reads) in at least one of the four samples were combined and represented as “other classified.” The table at the bottom shows, for each sample, the total number of reads, the percentage of classified reads, the number of classified reads classified as *Listeria* spp., and the proportion of classified reads classified as *Listeria* spp.

The genus level results from the shotgun sequencing analysis shown in Figure 7 corresponded well with the data from 16S rRNA gene amplicon sequencing (Figure 3 and Supplementary Figure 1). For *Pseudomonas*, amplicon sequencing yielded relative abundances of 11%, 10%, 57% in samples collected prior to enrichment and at t4 and t24 during primary enrichment. The corresponding values from the shotgun metagenomic data were 13%, 12%, and 70%. The most dominant *Pseudomonas* species were *Pseudomonas extremaustralis* and *Pseudomonas lurida*, reaching 34% and 19%, respectively, of the reads classified to the *Pseudomonas* genus after 24 h of primary enrichment.

For *Listeria*, however, 16S rRNA gene amplicon sequencing yielded relative abundances of 0.08%, 0.10%, and 2.0% in these three samples, while shotgun metagenomics yielded notably lower values of 0.0019%, 0.0024%, and 0.01%. Of the 26 reads mapping to *Listeria* from shotgun sequencing of the sample collected prior to enrichment, 22 mapped specifically to the species *L. monocytogenes*.

Considering the total bacterial count in the sample prior to enrichment (7 × 10^9^ TVC in 100 mL analyzed sample), the number of *Listeria* cells in the drain sample was estimated to 5.6 × 10^6^ cfu per drain sample based on the relative abundance determined by the 16S rRNA gene amplicon sequencing, and 1.3 × 10^5^ cfu per drain sample based on the shotgun sequencing data (1.6 log difference). For *Pseudomonas*, the corresponding values were slightly below 10^9^ cfu per drain sample using both datasets (0.1 log difference). Assuming that the concentration of *L. monocytogenes* in the primary enrichment culture did not decrease during the initial 4 h of enrichment, and that the *Listeria* population was composed primarily of *L. monocytogenes*, these numbers can be compared with that obtained by plating on selective agar after 4 h of enrichment. Here, two colonies were obtained, and the detection limit was 10^3^ cfu per sample, giving an estimated number of *L. monocytogenes* in the drain prior to enrichment of ≤2 × 10^3^ cfu per sample. Both estimates based on sequencing data were substantially higher than this (3.5 log and 1.8 log higher), suggesting that they overestimated the relative abundance of *Listeria*.

For the secondary enrichment sample (t48), 16S rRNA gene amplicon sequencing indicated the presence of 74% *Listeria* and 24% *Enterococcus*, while the corresponding values obtained with the quasimetagenomics approach were 50% and 46%. *Enterococcus* is a major source of false positive test results with rapid swab tests for detection of *Listeria* spp. (Schirmer et al., 2012). The majority of the *Enterococcus* shotgun sequencing reads mapped either to *Enterococcus* sp. CR-Ec1 (10%), *Enterococcus gallinarum* (9%), *Enterococcus* sp. FDAARGOS_375 (7%), or *Enterococcus casseliflavus* (9%). Of the reads classified as *Listeria* spp., 2.0 million reads were classified as *L. monocytogenes* (93%), 13 056 as *Listeria innocua* (0.6%), 10,496 as *Listeria welshimeri* (0.5%), and 396 as other named *Listeria* spp. (*Listeria grayi*, *Listeria seeligeri*, *Listeria ivanovii*, and *Listeria weihenstephanensis*).

### 3.8 MLST and strain-level typing of shotgun sequencing data

The two *L. monocytogenes* colonies obtained for drain E in week 1 by plating on selective agar after 4 h of primary enrichment were identified as CC9. The 10 colonies picked after 24 h primary enrichment in Half Fraser and after the subsequent 24 h secondary enrichment in Full Fraser were identified as CC9 (*n* = 9 and *n* = 8, respectively) and CC11-ST451 (*n* = 1 and *n* = 2, respectively) (Figure 6). According to these results, the drain E week 1 enrichment culture was dominated by CC9 (∼85%) with a minor proportion of CC11-ST451 (∼15%).

To examine whether similar results could be obtained directly from the metagenomic or quasimetagenomic sequencing data, the data was analyzed using MetaMLST, which is designed to identify the dominant MLST profile in a sample by read mapping to a database of MLST alleles (Zolfo et al., 2017). MetaMLST detected ST9, belonging to CC9, with 100% confidence in the sample from the secondary enrichment in Full Fraser broth. Allele read depths were reported to range from 277x to 424x. As expected, due to few *Listeria* reads in the samples from the drain or from the primary enrichment, no ST was detected by MetaMLST in these samples.

As an alternative MLST subtyping approach, the *Listeria* spp. and *L. monocytogenes* reads from of each Illumina shotgun sequencing sample were mapped to the Institute Pasteur’s *L. monocytogenes* MLST database using the KMA (*k*-mer alignment) method (Clausen et al., 2018). As for MetaMLST, no hits in the MLST profile database were obtained for the samples from the drain or from the primary enrichment. For the sample from the secondary enrichment, 15 MLST alleles were identified as perfect matches (100% identity and 100% coverage) by KMA (Supplementary Table 2). Seven alleles had read depths in the range 290x–430x, while the remaining alleles had read depths of 43x or lower. The seven alleles with read depths > 290x all had perfect matches in the KMA analysis, and corresponded to MLST profile 6-5-6-4-1-4-1, sequence type ST9. Furthermore, *bglA* allele 176, also a perfect match (read depth 4x), had exclusively been identified in ST1164, also belonging to CC9. This part of the analysis aligns with the output from MetaMLST.

Five of the eight remaining alleles with perfect matches identified in the KMA analysis had exclusively been identified in *L. innocua* and *L. welshmeri* strains (*abcZ* alleles 25 and 462, *bglA* allele 82, *dat* allele 20 and *ldh* allele 20). The last two perfect matches were *abcZ* allele 7 (read depth 11x) and *cat* allele 10 (read depth 43x). When examining all the possible MLST profile combinations containing these two alleles plus *bglA*, *dapE*, *dat*, *ldh* and *lhkA* alleles – including alleles that were not perfect matches – three possible MLST profiles were identified. All three belonged to CC11, and were ST451 with MLST profile 7-5-10-21-1-4-1, ST1558 with MLST profile 7-5-10-21-127-4-1 and ST2088 with MLST profile 7-332-10-21-1-4-1. The *dapE* allele 21 (present in all of these three STs) had a read depth of 6x, the *bglA* allele 332 had read depth 4x, while the *dat* allele 127 had read depth 1.4x. The percentage match (for both identity and coverage) for these three alleles were 96%, 99%–100%, and 76%, respectively.

Finally, the data was analyzed using the strain-typing tool StrainScan (Liao et al., 2023). This tool clusters a set of selected reference genomes based on *k*-mer Jaccard similarity and uses a hierarchical *k*-mer based indexing structure to compare sequencing reads to the reference genomes within each cluster. A database was built using one representative genome from each of the six CCs identified in the meat processing factory (CC9, CC11-ST451, CC121, CC8, CC199, and CC14-ST91), which resulted in six cluster search trees, each containing one strain. When running StrainScan with default settings, the relative abundances of *L. monocytogenes* strains in the secondary enrichment culture was predicted to be 85% of the representative CC9 genome and 15% of the representative CC11-ST451 genome. Predicted read depths were 165x and 29x, respectively. Notably, slightly different results were obtained when the database was built using the genomes of all 72 sequenced isolates from the processing factory (listed in Supplementary Table 1). In this case, the secondary enrichment culture was predicted to contain 94% CC9 and 6% CC11-ST451. The bias in representation of the two CC groups in this database (50 CC9 genomes and only three CC11-ST451 genomes) potentially influenced the outcome. No clusters were detected in the drain or primary enrichment samples when running StrainScan with default settings.

StrainScan was also run using settings that adjust the filtering cutoffs of the *k*-mer tree to better accommodate low sequencing depths, with a trade-off of lower confidence predictions. In this mode, the program detected 75% CC9, 13% CC11-ST451, 9% CC8, and 2% CC199 in the secondary enrichment culture, with associated read depths of 165x, 29x, 21x, and 5x, respectively. Additionally, for the 24 h primary enrichment sample, the program detected 17% CC9 and 83% CC8, with read depths of 1x and 5x, respectively. These results did not align with the MLST subtyping of single isolates (Figure 6), however, they indicated the possible presence of additional CCs at very low prevalence.

Overall, the data showed that corresponding results were obtained by picking and subtyping single colonies after primary or secondary enrichment and performing metagenomic sequencing of the secondary enrichment culture. All applied approaches showed that the secondary enrichment culture was dominated by CC9. Analysis using KMA and StrainScan was also able to predict a minor proportion of CC11 strains of (or related to) ST451.

## 4 Discussion

### 4.1 *L. monocytogenes* genetic diversity and persistence

Four different *L. monocytogenes* CCs were identified in the 24 samples from six floor drains examined in the current study (CC9, CC11-ST451, CC121, and CC8). Different CCs dominated in the different drains, and this remained consistent during the examined period of 9 weeks. The dominating CC appeared to correlate with the proximity of drains and whether the drains were wet or dry: Drains A, B, and F were in close proximity, all were usually dry and received limited levels of soiling and effluent, and all were dominated by CC11-ST451 isolates, with only a minority of CC9 detected. In contrast, drains C, D, and E, which were located in another part of the same processing area, were usually humid, dominated by CC9 isolates, but with higher diversity than the other drains. Three or four CCs were detected in each of these drains during the course of the monitoring period. Interestingly, the overall microbial richness (Shannon diversity) was significantly higher in the humid than the dry drains, mirroring the diversity of the CCs.

It is not necessarily evident whether a *L. monocytogenes* strain persists in a drain or whether the drain acts as a collector site for strains that persist in other niches in a facility. Potentially, drains in close proximity are more likely to be exposed to the same contamination source, e.g., during cleaning. An unknown factor in the current study was also whether or not any of the examined drains were directly connected by drainpipes beneath the floor. However, the observed stability of the microbial composition (both on genus and CC level) probably indicates that the drains represent a stable niche in which *L. monocytogenes* can persist. In either case, floor drains, along with other floor-associated sampling points, are important hygiene indicator sites for monitoring of *L. monocytogenes* in food processing facilities (Dzieciol et al., 2016; Simmons and Wiedmann, 2018).

### 4.2 Detection and diversity during selective enrichment

In three of the six examined drains, up to three co-occurring CCs were detected in the same sample. In some cases, different minority CCs were detected after primary and secondary enrichment. This underscores the importance of selecting more than one isolate for subtyping during investigation of contamination routes or persistence, as subtyping of only one single isolate from each sample may result in erroneous conclusions, e.g., during source tracking (Döpfer et al., 2008; Chen et al., 2020; Stessl et al., 2020; Wagner et al., 2021; Acciari et al., 2022).

*L. monocytogenes* was detected by plating on selective agar without prior selective enrichment in one-third of the examined samples. Culture-independent metagenomic shotgun sequencing also detected *L. monocytogenes* reads prior to enrichment. However, few colonies and very few shotgun sequencing reads were retrieved without enrichment. This approach was therefore not suited to capturing the diversity of CCs in the samples. Equivalent results were obtained for samples collected after 4 h of primary enrichment, in line with the limited growth and shift in the microbiota observed during the first 4 h of primary enrichment. Given the low number of shotgun sequencing reads classified as *L. monocytogenes* prior to enrichment, the higher values of relative abundance of *Listeria* in the drains estimated from sequencing data compared to from culturing methods, and known technical issues related to quantifying low abundance species in metagenomics data (further discussed below), it is challenging to ascertain whether *L. monocytogenes* was truly detected by direct metagenomic sequencing of drain microbiomes from naturally contaminated drains prior to selective enrichment.

The metagenomic approach applied to the secondary enrichment culture, which had a high relative abundance of *L. monocytogenes*, successfully detected both CCs identified by subtyping of 22 single isolates from the same culture. Additionally, this approach matched the relative abundances of CC9 and CC11-ST451 found by isolate subtyping. To capture diversity and the correct relative abundances of CCs, it is therefore advisable to pick and subtype multiple single colonies after 24 h of primary enrichment, or perform metagenomic sequencing on secondary enrichment cultures.

Analysis of metagenomic sequence data is still challenging, and requires selecting appropriate methods depending on the research question and dataset (Anyansi et al., 2020). An assembly-based approach involving metagenome-assembled genomes (MAGs) has been used for phylogenetic analysis of *L. monocytogenes* in food processing environments, but this method is unsuitable for multi-strain samples and requires high sequencing depth (Kocurek et al., 2023). In this study, the aim was to identify multiple MLST subtypes, including low-abundance subtypes, in multi-strain samples. For this purpose, alignment-based methods were selected, in which sequencing reads are aligned to reference databases. Two MLST database mapping approaches were used. While only the most dominant subtype was identified using MetaMLST (Zolfo et al., 2017), two different subtypes could be inferred from the KMA analysis output (Clausen et al., 2018) based on differential read depth coverage for each MLST allele. An algorithm integrating allele combinations (STs), CC prevalence data, and read depth would have simplified the analysis of the KMA output. Nevertheless, both MLST-based methods require significant sequencing depth, as they utilize only a small portion of the *L. monocytogenes* metagenome data.

Full genome alignment-based methods aim to differentiate metagenomic sequencing data at the strain level by aligning reads to a reference database of complete genomes, and assigning strains to the closest reference using probabilistic models (Anyansi et al., 2020). StrainScan, used in this study, organizes reference genomes into a hierarchical cluster tree and assigns metagenomic data to the nearest cluster, and within the cluster, to the nearest reference, using a *k*-mer based search strategy (Liao et al., 2023). The definition of a strain is, however, not clear-cut (Lindner et al., 2024), and assigning to a reference genome does not inherently solve assignment to a MLST subtype. The analysis requires that the CC type in the metagenome matches a CC in the reference database, allowing inference of the subtype. However, with adequate database representation, clustering methods like StrainScan are expected to work well for the species *L. monocytogenes*, as CCs largely correspond to clear and well-separated sublineages defined by phylogenetic analyses (Chen et al., 2016; Moura et al., 2016). Accordingly, StrainScan’s reference genome clusters showed a one-to-one correspondence with the six CCs included in the reference databases in the current study. Further analysis is, however, needed to confirm this for all *L. monocytogenes* CCs. The authors of StrainScan reported that the program showed high specificity and sensitivity in assigning strains down to 1x read depth (Liao et al., 2023). However, our findings indicate limitations in estimating the relative abundances of low-abundance strains and potential biases in these abundance estimates introduced by the composition of the reference database.

Estimates of absolute abundances, based on total bacterial counts, suggested that the 16S rRNA gene amplicon and shotgun metagenomics sequencing data overestimated the relative abundance of *Listeria* in the drains compared to values obtained from plating on *L. monocytogenes* selective agar plates. Such overestimation could be due to “sample bleeding,” i.e., incorrect assignment of an index (barcode) to a sequence from an adjacent cluster on the flow cell (Mitra et al., 2015). These issues are particularly noticeable in sequencing runs that include libraries with low sequence diversity, which was the case for most of the secondary enrichment samples in this study, which almost exclusively contained *Listeria*. Furthermore, the 16S rRNA gene amplicon sequencing approach, which used single barcodes, was expected to be more affected compared to the shotgun sequencing approach, which used dual barcodes. Quantification of taxa-specific abundances in microbial communities using sequencing based methods are known to be subject to various errors limiting direct comparison with culture-based quantifications, including DNA extraction efficiency, PCR-associated bias, variation in 16S rRNA operon copy numbers per genome, and inability to easily distinguish between viable and non-viable cells (Bonk et al., 2018). These errors can lead to significant deviations from true values, especially for low-abundance taxa (< 10%) (Tettamanti Boshier et al., 2020).

### 4.3 Molecular subtyping to CC level using qPCR

The Geno*Listeria* Multiplex qPCR scheme (Félix, 2023; Félix et al., 2023) has been referred to as a frontline screening tool for *L. monocytogenes* CCs, suitable for initial identification prior to selection of isolates for in-depth characterization using WGS, in the event of e.g., outbreaks (Jashari et al., 2024). The method was also a well-suited and cost-effective approach for the current study, where 460 isolates were subtyped. Sequential identification of CCs and selection of relevant qPCR reactions, based on previously identified CCs in the sampled environment, enabled the identification of 93.5% of the isolates in the dataset using approximately twice as many qPCR reactions as there were isolates to be subtyped. In our hands, the total costs (time cost plus direct costs) of strain typing would have been around six-fold more expensive had WGS been run for all DNA samples, compared with the combined qPCR / WGS approach. The use of the method, however, requires careful consideration of the chosen amplification cycle threshold (Ct) that differentiates positive and negative analysis results. The original method indicates that reactions with Ct ≤ 25 should be considered positive (Félix, 2023; Félix et al., 2023), while another study set the Ct cutoff at ≤ 30 (Jashari et al., 2024). In the present work, amplifications with Ct < 18 were considered positive, and in several cases, calling detections with Ct values in the range 20–25 as positive would have given erroneous results.

The genetic marker *lmo1118* used to detect CC9 in the Geno*Listeria* scheme (Félix, 2023; Félix et al., 2023) is the same as the marker used to identify PCR-serogroup IIc (Doumith et al., 2004; Vitullo et al., 2013). Félix et al., 2023 reported that 3% of CC9 genomes in a panel comprising 142 CC9 isolates lacked *lmo1118*, while no false-negative results were reported for CC9 (*n* = 38) in another report (Jashari et al., 2024). In the current study, 12% of all typed CC9 isolates (*n* = 212), and 32% (*n* = 22) of CC9 isolates previously identified by WGS from the same meat processing factory (Fagerlund et al., 2022; Ivanova et al., 2025), lacked the *lmo1118* marker. Potentially, it is possible to identify a more suitable molecular target for CC9. In comparison, an *in silico* analysis of 1241 Norwegian *L. monocytogenes* isolates using the GENE-UP Typer from bioMérieux was able to correctly identify 99.7% of the 313 CC9 isolates included in the analysis (Fagerlund et al., 2024). This method uses a kit with eight duplex qPCR reactions comprising 16 target marker genes, followed by analysis using probabilistic algorithms to predict the subtype based on an underlying WGS-based phylogeny.

Although WGS is the gold standard subtyping method (Lakicevic et al., 2023), new simpler methods such as Geno*Listeria* Multiplex and GENE-UP Typer continue to emerge, suggesting that there is still a need for alternative genotyping methods for pathogens such as *L. monocytogenes*. This likely reflects current challenges related to the cost and analysis time of WGS. Ideally, an alternative subtyping method should be fast, practical, and able to distinguish known genetic groups identified from WGS-based studies (i.e., CCs for *L. monocytogenes*) with high sensitivity and specificity. Compared to methods like PFGE and MLVA, PCR-based assays have the advantage of being concordant with *in silico* predictions of subtyping results from WGS data, also when WGS is performed using short read sequencing technology (Kwong et al., 2016). This is particularly relevant when a combination of methods is used, such as using simple genotyping on many (or most) isolates and WGS on a selection or for untypeable isolates, or when there is a need to compare results with externally available WGS data. In this context, PCR-based genotyping methods represent a sustainable option until technological developments or increased usage reduce the cost and analysis time for WGS. Additionally, it would be interesting to explore whether PCR-based genotyping is suitable for analyzing DNA obtained directly from enrichment culture samples and if it can identify more than one CC present in a sample.

### 4.4 Microbial composition and association with *L. monocytogenes*

Floor drains are colonized by different microbes that can persist over time despite cleaning and disinfection (Dzieciol et al., 2016; Belk et al., 2022). This resident microbiota consists mainly of non-pathogenic bacteria but may also be a reservoir for pathogens like *L. monocytogenes* (Fagerlund et al., 2021), and several studies have identified drains as common sites for persistent *L. monocytogenes* (Burnett et al., 2020; Belias et al., 2022; EFSA Panel on Biological Hazards (BIOHAZ) et al., 2024). There is ongoing debate regarding the association between the microbial composition and the presence or absence of *L. monocytogenes* in samples or biofilms from the food industry. For example, it has been shown that *L. monocytogenes* can be established in *Pseudomonas* biofilms (Fagerlund et al., 2021), while other studies have shown lower abundance of specific *Pseudomonas* species in *Listeria*-positive compared to *Listeria*-negative samples (Pracser et al., 2024). A wide range of biofilm producers have been associated with the presence of *Listeria* spp. in food processing plants, including the most abundant genera observed in drains in the current study; i.e., *Pseudomonas*, *Acinetobacter* and *Janthinobacterium* (Zwirzitz et al., 2021; Belk et al., 2022; Pracser et al., 2024). The presence of *Janthinobacterium* has, however, also been associated with lower prevalence of *L. monocytogenes* positive drains (Fox et al., 2014).

*Pseudomonas* was the most abundant and consistently found genus in the majority of drains in the current study. It is frequently found in food processing plants (Møretrø and Langsrud, 2017). It was also recently reported to be the most abundant and stable component of the microbiota on floor surfaces and drain in a RTE meat factory (Diaz et al., 2025), and in drains in a small meat production facility (Belk et al., 2022). Both studies found that the drain microbiota composition remained relatively stable over time.

Since *Pseudomonas* is commonly found on both raw materials and food contact surfaces (Møretrø and Langsrud, 2004), its presence in drains is likely not significant for the food quality of the final product. In this study, all drains were positive for *L. monocytogenes* in at least one sampling week, while Diaz et al. (2025) found few *L. monocytogenes* positive samples overall. Although it has been suggested that *Pseudomonas* may affect the growth and survival of *L. monocytogenes* in biofilms (Fagerlund et al., 2021), our study does not support this claim, as no clear association between resident drain microbiota and the presence or absence of *L. monocytogenes* was found for either study.

Drains are among the most common harbourage locations for *Listeria* spp. in food processing facilities. Current monitoring approaches may underestimate the persistence of the same clone over time. Therefore, implementing methodology capable of detecting multiple clones within shorter timeframes should be encouraged. Corrective actions beyond regular cleaning and disinfection should be initiated in case of repeated detection of *L. monocytogenes* in drains. According to guidelines for food processers, extended exposure time (overnight) of cleaning agents or disinfectants combined with brushing may resolve issues with persistent *L. monocytogenes* (Fagerlund et al., 2020, Tuytschaever et al., 2023).

## 5 Conclusion

The current study highlights the use of alternative techniques such as qPCR and quasimetagenomics, which may be more cost-effective and rapid, for monitoring and mitigating the risk of contamination in processing environments. Despite being the method with highest sensitivity and specificity, WGS may not always be practical, e.g., when multiple subtypes of *L. monocytogenes* are present in the same sample. In that regard, the study demonstrated that shotgun metagenomic sequencing of a secondary enrichment culture provided information about the relative abundance of *L. monocytogenes* clonal complexes (CCs) in a sample, comparable to the information obtained from identifying multiple single isolates after primary or secondary enrichment. It should be noted, however, that results from both metagenomic sequencing analyses and the Geno*Listeria* Multiplex qPCR may be challenging to interpret, taking into account e.g., potential biases and errors in metagenomic sequencing and data processing, and challenges with lack of genetic markers and selection of Ct cutoffs for qPCR reactions.

In conclusion, this study provides valuable insights into the microbiota and population dynamics of *L. monocytogenes* in drains in a meat processing environment. The persistence and genetic diversity of *L. monocytogenes* underscore the importance of implementing new tools for surveillance of this pathogen in food processing facilities. Future research should focus on further elucidating the interactions between *L. monocytogenes* and the resident microbiota, as well as developing and validating more effective monitoring techniques to mitigate the risk of *L. monocytogenes* contamination in the food supply chain.

## Data Availability

The datasets presented in this study can be found in online repositories. The names of the repository/repositories and accession number(s) can be found in the article/Supplementary material.

## Footnotes

1
https://www.biomerieux.com/corp/en/our-offer/industry-products/gene-up-typer.html

2
https://rheonix.com/food-beverage-testing/listeria-patternalert-assay/

3
https://bigsdb.pasteur.fr/listeria/
