# Supplementary material for: Early detection and population dynamics of Listeria monocytogenes in naturally contaminated drains from a meat processing plant
Source: Front Microbiol. 2025 Apr 9;16:1541481. doi: 10.3389/fmicb.2025.1541481 (PMC12014604; doi:10.3389/fmicb.2025.1541481)
Supplement: Supplementary file 1 [file Data_Sheet_1.pdf]

Supplementary Information

Early detection and population dynamics of *Listeria monocytogenes* in naturally contaminated drains from a meat processing plant

Annette Fagerlund, Trond Møretrø, Merete Rusås Jensen, Solveig Langsrud, and Birgitte Moen

Nofima, Norwegian Institute of Food, Fisheries and Aquaculture Research, Department of Food Safety and Quality, N-1430 Ås, Norway

Frontiers in Microbiology, 2025

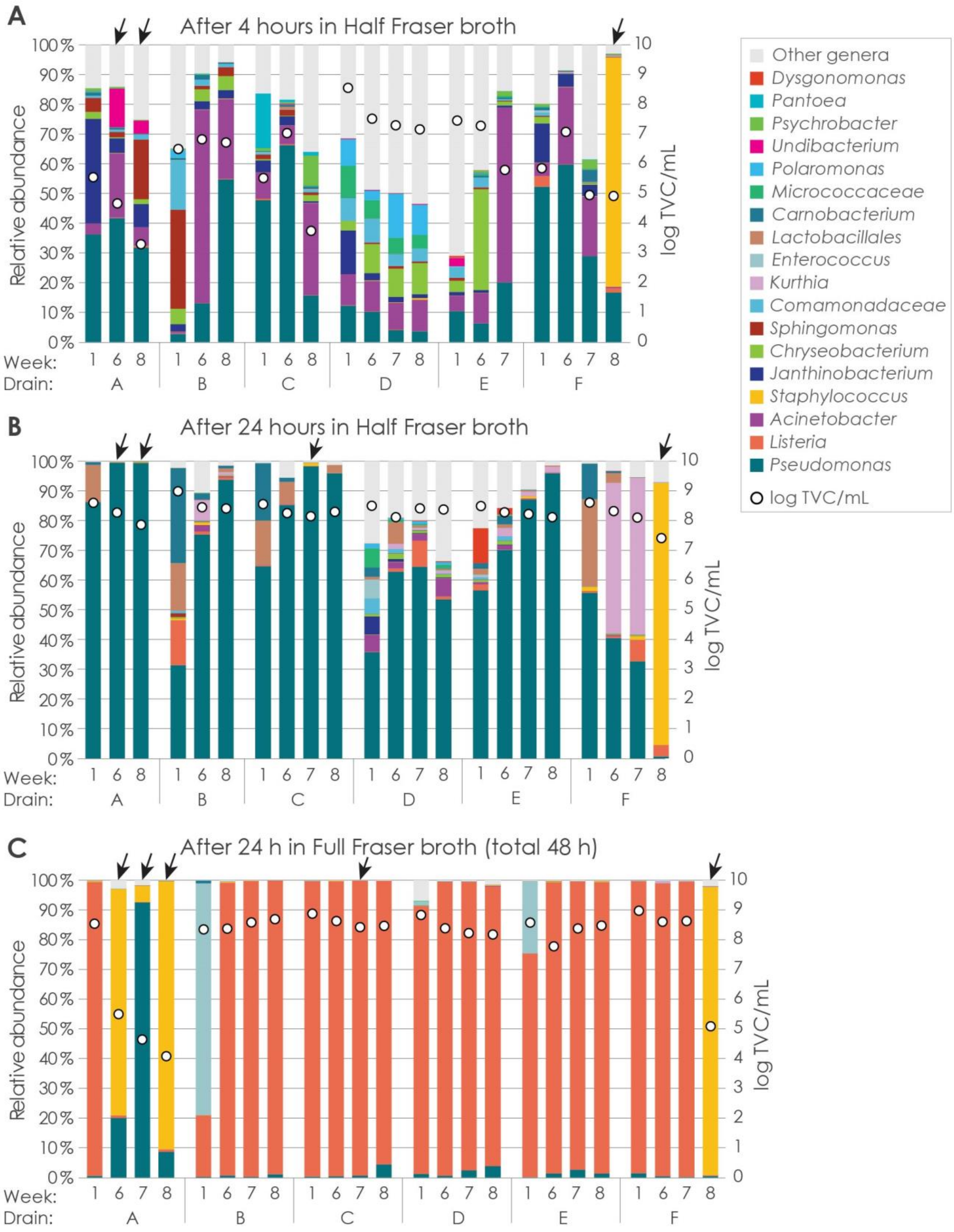

Figure S1: Relative abundance (%) of bacterial genera during selective enrichment. Samples were from **(A)** after 4 hours and **(B)** after 24 hours of primary enrichment in Half Fraser broth, and **(C)** after 24 hours of secondary enrichment in Full Fraser broth. Samples negative for *L. monocytogenes* are indicated by arrows. See Figure 3 legend for further description.

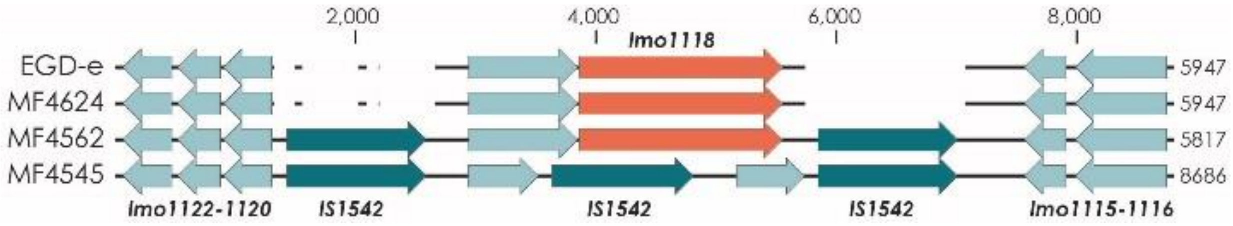

Figure S2: Alignment of the genomic location surrounding *lmo1118* in EGD-e and in six completely sequenced Norwegian isolates. The *lmo1118* gene is shown in red, and the IS*1542* insertion sequence is shown in dark blue. MF4626, MF4697, and MF6172 were identical to MF4624 and EGD-e and are not shown.

Table S1: List of WGS genomes

Name	Collection Year	Sample type	Drain	Week	sample	No	MLST	BioProject	BioSample	SRA Run	GenBank assembly accession number	Reference
MF8691	2022	Drain	A	1	t24	1	ST451	PRJNA1088322	SAMN40504363	SRR28386559	GCA_044442145.1	current study
MF8694	2022	Drain	B	9	FF	4	ST9	PRJNA1088322	SAMN40504366	SRR28386556	GCA_044442085.1	current study
MF8687	2022	Drain	B	9	FF	6	ST9	PRJNA1088322	SAMN40504359	SRR28386564	GCA_044442185.1	current study
MF8364	2022	Drain	B	9	t24	3	ST9	PRJNA1088322	SAMN40467751	SRR28360472	GCA_044440805.1	current study
MF8686	2022	Drain	B	9	t24	6	ST9	PRJNA1088322	SAMN40504358	SRR28386565	GCA_044443045.1	current study
MF8693	2022	Drain	C	7	t24	2	ST121	PRJNA1088322	SAMN40504365	SRR28386557	GCA_044442065.1	current study
MF8357	2022	Drain	D	1	t4	1	ST9	PRJNA1088322	SAMN40467744	SRR28360479	GCA_044441025.1	current study
MF8689	2022	Drain	D	1	t4	9	ST9	PRJNA1088322	SAMN40504361	SRR28386562	GCA_044442205.1	current study
MF8361	2022	Drain	D	7	FF	6	ST8	PRJNA1088322	SAMN40467748	SRR28360475	GCA_044440825.1	current study
MF8688	2022	Drain	D	9	FF	9	ST9	PRJNA1088322	SAMN40504360	SRR28386563	GCA_044442165.1	current study
MF8365	2022	Drain	D	9	t24	8	ST9	PRJNA1088322	SAMN40467752	SRR28360471	GCA_044440745.1	current study
MF8358	2022	Drain	E	1	t24	10	ST9	PRJNA1088322	SAMN40467745	SRR28360478	GCA_044440965.1	current study
MF8673	2022	Drain	E	7	FF	1	ST9	PRJNA1088322	SAMN40504345	SRR28386560	GCA_044442625.1	current study
MF8674	2022	Drain	E	7	FF	2	ST9	PRJNA1088322	SAMN40504346	SRR28386555	GCA_044442605.1	current study
MF8675	2022	Drain	E	7	FF	3	ST9	PRJNA1088322	SAMN40504347	SRR28386554	GCA_044442505.1	current study
MF8676	2022	Drain	E	7	FF	5	ST9	PRJNA1088322	SAMN40504348	SRR28386553	GCA_044442525.1	current study
MF8677	2022	Drain	E	7	FF	6	ST9	PRJNA1088322	SAMN40504349	SRR28386552	GCA_044442545.1	current study
MF8678	2022	Drain	E	7	FF	7	ST9	PRJNA1088322	SAMN40504350	SRR28386551	GCA_044442385.1	current study
MF8679	2022	Drain	E	7	FF	8	ST9	PRJNA1088322	SAMN40504351	SRR28386550	GCA_044442425.1	current study
MF8360	2022	Drain	E	7	t24	1	ST9	PRJNA1088322	SAMN40467747	SRR28360476	GCA_044440945.1	current study
MF8672	2022	Drain	E	7	t24	10	ST9	PRJNA1088322	SAMN40504344	SRR28386571	GCA_044442645.1	current study
MF8359	2022	Drain	E	7	t4	2	ST9	PRJNA1088322	SAMN40467746	SRR28360477	GCA_044440885.1	current study
MF8671	2022	Drain	E	7	t4	3	ST9	PRJNA1088322	SAMN40504343	SRR28386572	GCA_044442765.1	current study
MF8363	2022	Drain	E	8	FF	1	ST8	PRJNA1088322	SAMN40467750	SRR28360473	GCA_044440725.1	current study
MF8680	2022	Drain	E	8	FF	3	ST9	PRJNA1088322	SAMN40504352	SRR28386549	GCA_044442405.1	current study
MF8681	2022	Drain	E	8	FF	5	ST8	PRJNA1088322	SAMN40504353	SRR28386570	GCA_044442325.1	current study
MF8682	2022	Drain	E	8	FF	6	ST9	PRJNA1088322	SAMN40504354	SRR28386569	GCA_044442345.1	current study
MF8683	2022	Drain	E	8	FF	8	ST9	PRJNA1088322	SAMN40504355	SRR28386568	GCA_044442285.1	current study
MF8684	2022	Drain	E	8	FF	9	ST9	PRJNA1088322	SAMN40504356	SRR28386567	GCA_044442245.1	current study
MF8685	2022	Drain	E	8	FF	10	ST9	PRJNA1088322	SAMN40504357	SRR28386566	GCA_044443165.1	current study
MF8362	2022	Drain	E	8	t24	1	ST9	PRJNA1088322	SAMN40467749	SRR28360474	GCA_044440845.1	current study
MF8690	2022	Drain	E	8	t24	3	ST9	PRJNA1088322	SAMN40504362	SRR28386561	GCA_044442125.1	current study
MF8366	2022	Drain	E	9	FF	6	ST9	PRJNA1088322	SAMN40467753	SRR28360470	GCA_044440685.1	current study
MF8692	2022	Drain	F	8	t0	1	ST451	PRJNA1088322	SAMN40504364	SRR28386558	GCA_044442105.1	current study
MF7318	2017	other					ST9	PRJNA689484	SAMN17224462	SRR13588320	GCA_024251425.1	(1)
MF7320	2017	other					ST9	PRJNA689484	SAMN17224463	SRR13588323	GCA_024251445.1	(1)
MF7321	2017	Drain	other				ST9	PRJNA689484	SAMN17224464	SRR13588319	GCA_024251405.1	(1)
MF7324	2017	Drain	other				ST9	PRJNA689484	SAMN17224465	SRR13588318	GCA_024251345.1	(1)
MF7325	2017	other					ST121	PRJNA689484	SAMN17224466	SRR13588317	GCA_024251385.1	(1)
MF7326	2017	other					ST9	PRJNA689484	SAMN17224467	SRR13588316	GCA_024251355.1	(1)
MF7403	2018	Drain	other				ST9	PRJNA689484	SAMN17224507	SRR13588294	GCA_024250525.1	(1)
MF7408	2018	other					ST199	PRJNA689484	SAMN17224508	SRR13588293	GCA_024250605.1	(1)
MF7413	2019	other					ST121	PRJNA689484	SAMN17224509	SRR13588292	GCA_024250505.1	(1)
MF7416	2019	Drain	other				ST9	PRJNA689484	SAMN17224510	SRR13588291	GCA_024250485.1	(1)
MF7420	2019	other					ST121	PRJNA689484	SAMN17224511	SRR13588290	GCA_024250455.1	(1)
MF7421	2019	Drain	other				ST9	PRJNA689484	SAMN17224512	SRR13588289	GCA_024250435.1	(1)
MF7426	2019	Drain	other				ST9	PRJNA689484	SAMN17224513	SRR13588287	GCA_024250405.1	(1)
MF7427	2019	Drain	other				ST9	PRJNA689484	SAMN17224514	SRR13588286	GCA_024250425.1	(1)
MF7429	2019	other					ST9	PRJNA689484	SAMN17224515	SRR13588285	GCA_024250375.1	(1)
MF7430	2019	other					ST9	PRJNA689484	SAMN17224516	SRR13588284	GCA_024250365.1	(1)
MF7432	2019	other					ST9	PRJNA689484	SAMN17224517	SRR13588283	GCA_024250315.1	(1)
MF7437	2019	Drain	other				ST9	PRJNA689484	SAMN17224518	SRR13378886	GCA_024250305.1	(1)
MF7648	2019	other					ST451	PRJNA689484	SAMN17224522	SRR13588136	GCA_024250265.1	(1)
MF7649	2019	other					ST199	PRJNA689484	SAMN17224523	SRR13588135	GCA_024250215.1	(1)
MF7650	2019	Drain	other				ST199	PRJNA689484	SAMN17224524	SRR13588134	GCA_024250205.1	(1)
MF7651	2019	other					ST121	PRJNA689484	SAMN17224525	SRR13588133	GCA_024250185.1	(1)
MF7654	2019	Drain	other				ST121	PRJNA689484	SAMN17224526	SRR13588132	GCA_024250155.1	(1)
MF7657	2019	other					ST121	PRJNA689484	SAMN17224527	SRR13588334	GCA_024250145.1	(1)
MF7322	2017	Drain	other				ST121	PRJEB56155	SAMEA112171252	ERR10612076		(2)
MF7327	2017	Drain	other				ST91	PRJEB56155	SAMEA112171253	ERR10612074		(2)
MF7402	2018	Drain	D				ST9	PRJEB56155	SAMEA112171263	ERR10612088		(2)
MF7405	2018	Drain	D				ST9	PRJEB56155	SAMEA112171264	ERR10612080		(2)
MF7411	2018	Drain	D				ST121	PRJEB56155	SAMEA112171265	ERR10612108		(2)
MF7412	2018	Drain	other				ST9	PRJEB56155	SAMEA112171266	ERR10612121		(2)
MF7419	2019	Drain	D				ST121	PRJEB56155	SAMEA112171267	ERR10612130		(2)
MF7435	2019	Drain	other				ST199	PRJEB56155	SAMEA112171268	ERR10612090		(2)
MF7627	2019	Drain	D				ST9	PRJEB56155	SAMEA112171269	ERR10612122		(2)
MF7628	2019	Drain	other				ST9	PRJEB56155	SAMEA112171270	ERR10612111		(2)
MF7631	2019	Drain	D				ST9	PRJEB56155	SAMEA112171271	ERR10612141		(2)
MF7632	2019	Drain	other				ST9	PRJEB56155	SAMEA112171272	ERR10612120		(2)
MF7633	2019	Drain	other				ST121	PRJEB56155	SAMEA112171273	ERR10612110		(2)
MF7638	2019	Drain	other				ST9	PRJEB56155	SAMEA112171274	ERR10612114		(2)

Table S2: Mapping of reads to MLST database using KMA

Output from mapping of Illumina reads classified as *Listeria* spp. or *L. monocytogenes* from the quasimetagenomic sequencing of the secondary enrichment culture in Fraser broth (t48) for drain E in week 1. Perfect matches are indicated in color and bold.

#Template	Score	Expected	Template length	Template Identity	Template Coverage	Query Identity	Query Coverage	Depth	q_value	p_value
**abcZ_6**	**197763**	**430**	**537**	**100.00**	**100.00**	**100.00**	**100.00**	**370.96**	**196475.28**	**1.0e-26**
**abcZ_7**	**5692**	**494**	**537**	**100.00**	**100.00**	**100.00**	**100.00**	**10.67**	**4367.18**	**1.0e-26**
abcZ_16	21784	488	537	91.06	91.06	100.00	109.82	40.88	20360.18	1.0e-26
abcZ_21	560	495	537	91.06	91.25	99.80	109.59	1.09	3.88	4.9e-02
**abcZ_25**	**3922**	**494**	**537**	**100.00**	**100.00**	**100.00**	**100.00**	**7.34**	**2659.23**	**1.0e-26**
abcZ_26	567	495	537	74.86	75.05	99.75	133.25	1.09	4.75	2.9e-02
abcZ_54	891	495	537	76.91	77.28	99.52	129.40	1.72	112.59	1.0e-25
abcZ_71	579	495	537	58.10	58.10	100.00	172.12	1.09	6.42	1.1e-02
abcZ_73	1585	496	538	99.63	99.81	99.81	100.19	3.13	569.16	1.0e-26
abcZ_95	515	495	537	83.43	83.80	99.56	119.33	1.00	0.36	5.5e-01
abcZ_100	504	495	537	52.14	53.82	96.89	185.81	1.06	0.06	8.0e-01
abcZ_144	407	496	537	41.15	41.34	99.55	241.89	0.77	8.77	3.1e-03
abcZ_147	501	495	537	54.56	54.93	99.32	182.03	0.97	0.03	8.7e-01
abcZ_184	413	496	537	74.12	74.67	99.25	133.92	0.80	7.58	5.9e-03
abcZ_186	3524	494	537	70.58	70.58	100.00	141.69	6.63	2282.91	1.0e-26
abcZ_206	7863	493	537	60.34	60.34	100.00	165.74	14.31	6498.98	1.0e-26
abcZ_209	955	495	537	92.55	92.74	99.80	107.83	2.00	145.32	1.0e-26
abcZ_242	467	495	537	67.41	67.78	99.45	147.53	0.94	0.87	3.5e-01
abcZ_252	586	495	537	72.07	72.44	99.49	138.05	1.10	7.49	6.2e-03
abcZ_256	524	495	537	71.32	71.51	99.74	139.84	1.07	0.77	3.8e-01
abcZ_288	960	495	537	67.60	67.78	99.73	147.53	1.91	147.99	1.0e-26
abcZ_300	531	495	537	53.63	54.93	97.63	182.03	1.08	1.19	2.7e-01
abcZ_316	775	495	537	80.82	81.94	98.64	122.05	1.50	61.30	1.0e-14
abcZ_320	614	495	537	72.44	72.81	99.49	137.34	1.18	12.56	3.9e-04
abcZ_328	866	495	537	56.98	57.36	99.35	174.35	1.67	100.60	1.0e-22
abcZ_384	475	495	537	39.85	43.39	91.85	230.47	1.02	0.45	5.0e-01
abcZ_398	550	495	537	98.14	100.00	98.14	100.00	1.11	2.79	9.5e-02
abcZ_435	693	495	537	57.54	57.91	99.36	172.67	1.41	32.67	1.1e-08
**abcZ_462**	**1894**	**495**	**537**	**100.00**	**100.00**	**100.00**	**100.00**	**3.53**	**818.47**	**1.0e-26**
abcZ_480	570	495	537	75.42	75.79	99.51	131.94	1.11	5.14	2.3e-02
**bglA_5**	**157278**	**329**	**399**	**100.00**	**100.00**	**100.00**	**100.00**	**397.17**	**156291.31**	**1.0e-26**
bglA_15	1256	368	399	92.23	92.23	100.00	108.42	3.14	485.13	1.0e-26
bglA_64	697	368	399	99.50	100.00	99.50	100.00	1.85	101.32	1.0e-23
bglA_78	870	368	399	55.89	56.14	99.55	178.12	2.40	203.17	1.0e-26
**bglA_82**	**1470**	**368**	**399**	**100.00**	**100.00**	**100.00**	**100.00**	**3.71**	**660.33**	**1.0e-26**
bglA_121	507	368	399	66.42	68.42	97.07	146.15	1.33	21.91	2.9e-06
bglA_146	933	368	399	94.99	95.49	99.48	104.72	2.50	244.96	1.0e-26
bglA_147	327	368	399	75.19	75.69	99.34	132.12	0.85	2.48	1.2e-01
bglA_170	764	368	399	93.48	96.24	97.14	103.91	2.08	138.18	1.0e-26
**bglA_176**	**1332**	**368**	**399**	**100.00**	**100.00**	**100.00**	**100.00**	**3.65**	**546.23**	**1.0e-26**
bglA_209	667	368	399	94.99	95.74	99.21	104.45	1.88	86.08	1.0e-19
bglA_296	660	368	399	68.92	69.92	98.57	143.01	1.74	82.65	1.0e-19
bglA_332	1593	368	399	99.75	100.00	99.75	100.00	4.17	764.87	1.0e-26
bglA_423	427	368	399	72.68	74.69	97.32	133.89	1.21	4.30	3.8e-02
**cat_6**	**207111**	**386**	**486**	**100.00**	**100.00**	**100.00**	**100.00**	**429.88**	**205953.54**	**1.0e-26**
**cat_10**	**20587**	**442**	**486**	**100.00**	**100.00**	**100.00**	**100.00**	**42.78**	**19295.82**	**1.0e-26**
cat_15	403	448	486	59.26	59.47	99.65	168.17	0.85	2.47	1.2e-01
cat_17	788	448	486	70.99	71.40	99.42	140.06	1.87	93.04	1.0e-21
cat_28	582	448	486	46.09	46.09	100.00	216.96	1.24	17.20	3.4e-05
cat_30	797	448	486	63.17	63.17	100.00	158.31	1.65	97.34	1.0e-22
cat_37	830	448	486	70.58	71.60	98.56	139.66	1.83	113.66	1.0e-25
cat_58	1539	448	486	82.51	82.72	99.75	120.90	3.29	598.26	1.0e-26
cat_101	608	448	486	62.96	63.17	99.67	158.31	1.29	23.97	9.8e-07
cat_107	741	448	486	87.24	87.24	100.00	114.62	1.56	71.77	1.0e-16
cat_109	1447	448	486	99.79	100.00	99.79	100.00	3.32	525.88	1.0e-26
cat_111	818	448	486	88.89	88.89	100.00	112.50	1.78	107.62	1.0e-24
cat_114	651	448	486	41.36	41.77	99.01	239.41	1.46	37.17	1.6e-09

cat_122	730	448	486	77.16	77.57	99.47	128.91	1.63	67.08	1.0e-15
cat_131	1342	448	486	79.63	79.84	99.74	125.26	2.95	445.74	1.0e-26
cat_139	489	448	486	30.04	30.45	98.65	328.38	1.08	1.72	1.9e-01
cat_140	1257	448	486	83.74	85.19	98.31	117.39	2.76	383.11	1.0e-26
cat_147	887	448	486	66.26	66.46	99.69	150.46	2.02	143.79	1.0e-26
cat_191	1101	448	486	87.04	87.24	99.76	114.62	2.33	274.58	1.0e-26
cat_195	506	448	486	61.32	61.52	99.67	162.54	1.20	3.42	6.4e-02
cat_235	751	448	486	63.79	64.20	99.36	155.77	1.63	76.12	1.0e-17
cat_264	860	448	486	86.01	87.24	98.58	114.62	1.96	129.22	1.0e-26
cat_306	375	448	486	30.66	31.07	98.68	321.85	0.83	6.63	1.0e-02
cat_350	879	448	486	72.63	73.25	99.16	136.52	1.93	139.42	1.0e-26
cat_351	1266	448	486	99.59	99.59	100.00	100.41	2.62	389.64	1.0e-26
cat_373	698	448	486	57.61	57.82	99.64	172.95	1.63	54.15	1.0e-12
cat_375	492	448	486	38.27	38.68	98.94	258.51	1.07	1.98	1.6e-01
cat_420	848	448	486	70.58	70.58	100.00	141.69	1.79	122.92	1.0e-26
cat_441	401	448	486	70.58	72.02	98.00	138.86	0.91	2.70	1.0e-01
cat_452	1023	448	486	59.47	62.76	94.75	159.34	2.32	224.10	1.0e-26
cat_456	372	448	486	42.59	42.59	100.00	234.78	0.78	7.20	7.3e-03
**dapE_4**	**165194**	**379**	**462**	**100.00**	**100.00**	**100.00**	**100.00**	**360.41**	**164058.54**	**1.0e-26**
dapE_5	1137	426	462	75.97	75.97	100.00	131.62	2.67	322.86	1.0e-26
dapE_10	11275	423	462	82.90	82.90	100.00	120.63	24.68	10065.52	1.0e-26
dapE_21	2776	426	462	95.67	95.67	100.00	104.52	6.05	1724.62	1.0e-26
dapE_22	750	426	462	85.06	85.50	99.49	116.96	1.84	88.88	1.0e-20
dapE_29	18867	421	462	77.92	77.92	100.00	128.33	41.17	17639.50	1.0e-26
dapE_32	554	426	462	96.75	96.75	100.00	103.36	1.20	16.53	4.8e-05
dapE_55	511	426	462	59.96	60.82	98.58	164.41	1.19	7.58	5.9e-03
dapE_105	1002	426	462	27.06	27.27	99.21	366.67	2.15	231.81	1.0e-26
dapE_112	547	426	462	99.35	100.00	99.35	100.00	1.24	14.87	1.2e-04
dapE_116	547	426	462	67.10	68.18	98.41	146.67	1.22	14.87	1.2e-04
dapE_119	1456	426	462	99.78	100.00	99.78	100.00	3.26	563.13	1.0e-26
dapE_127	836	426	462	75.76	76.41	99.15	130.88	1.89	132.75	1.0e-26
dapE_129	569	426	462	99.13	100.00	99.13	100.00	1.29	20.35	6.5e-06
dapE_133	736	426	462	97.84	100.00	97.84	100.00	1.70	82.33	1.0e-18
dapE_134	518	426	462	74.24	75.11	98.85	133.14	1.16	8.83	3.0e-03
dapE_137	562	426	462	67.75	67.97	99.68	147.13	1.29	18.52	1.7e-05
dapE_178	443	426	462	98.27	100.00	98.27	100.00	1.01	0.31	5.8e-01
dapE_180	1015	426	462	87.45	88.31	99.02	113.24	2.46	240.22	1.0e-26
dapE_182	700	426	462	88.96	88.96	100.00	112.41	1.52	66.33	1.0e-15
dapE_186	574	426	462	68.18	68.40	99.68	146.20	1.29	21.69	3.2e-06
dapE_207	1113	426	462	83.77	83.77	100.00	119.38	2.42	306.11	1.0e-26
dapE_219	1266	426	462	90.04	90.48	99.52	110.53	2.99	416.44	1.0e-26
dapE_276	572	426	462	80.09	80.30	99.73	124.53	1.24	21.15	4.3e-06
dapE_279	683	426	462	88.74	90.04	98.56	111.06	1.55	59.23	1.0e-13
dapE_402	871	426	462	64.94	64.94	100.00	154.00	2.00	152.21	1.0e-26
dapE_428	908	426	462	75.54	75.54	100.00	132.38	2.19	173.67	1.0e-26
dapE_442	1482	426	462	95.45	96.10	99.32	104.05	3.40	583.87	1.0e-26
dapE_552	542	426	462	67.75	69.05	98.12	144.83	1.28	13.73	2.1e-04
dapE_567	747	426	462	71.43	71.65	99.70	139.58	1.73	87.46	1.0e-20
dapE_572	437	426	462	73.59	74.46	98.84	134.30	1.04	0.12	7.3e-01
dapE_575	933	426	462	94.37	94.59	99.77	105.72	2.17	188.64	1.0e-26
**dat_1**	**175016**	**384**	**471**	**100.00**	**100.00**	**100.00**	**100.00**	**373.55**	**173866.81**	**1.0e-26**
dat_5	668	434	471	80.04	81.53	98.18	122.66	1.62	49.24	1.0e-11
dat_8	510	435	471	73.25	73.89	99.14	135.34	1.25	5.95	1.5e-02
dat_9	825	434	471	99.15	100.85	98.32	99.16	1.87	120.78	1.0e-26
**dat_20**	**1372**	**434**	**471**	**100.00**	**100.00**	**100.00**	**100.00**	**2.92**	**486.20**	**1.0e-26**
dat_39	797	434	471	67.09	67.30	99.68	148.58	1.77	106.42	1.0e-24
dat_54	574	434	471	48.41	48.41	100.00	206.58	1.22	19.15	1.2e-05
dat_76	724	434	471	73.46	73.89	99.43	135.34	1.67	72.10	1.0e-16
dat_107	435	435	471	75.80	78.77	96.23	126.95	1.11	0.00	1.0e+00
dat_108	456	435	471	97.45	100.00	97.45	100.00	1.10	0.49	4.8e-01
dat_111	415	435	471	89.81	90.66	99.06	110.30	0.91	0.47	4.9e-01
dat_127	613	434	471	75.80	76.01	99.72	131.56	1.44	30.24	3.9e-08
dat_152	1523	434	471	99.79	100.00	99.79	100.00	3.34	604.98	1.0e-26
dat_178	528	434	471	77.71	78.77	98.65	126.95	1.20	8.98	2.7e-03
dat_183	907	434	471	94.48	94.69	99.78	105.61	2.15	166.10	1.0e-26
dat_193	1137	434	471	99.58	100.00	99.58	100.00	2.49	313.69	1.0e-26
dat_208	550	434	471	58.39	58.39	100.00	171.27	1.17	13.43	2.5e-04

dat_210	885	434	471	98.73	99.15	99.57	100.86	2.13	153.49	1.0e-26
dat_228	607	434	471	52.87	54.14	97.65	184.71	1.35	28.40	9.9e-08
dat_256	467	435	471	67.52	69.85	96.66	143.16	1.11	1.13	2.9e-01
dat_280	555	434	471	63.06	63.69	99.00	157.00	1.23	14.55	1.4e-04
dat_322	528	434	471	47.56	48.62	97.82	205.68	1.25	8.98	2.7e-03
dat_324	636	434	471	68.15	68.15	100.00	146.73	1.44	37.74	1.3e-09
dat_336	365	435	471	54.14	54.99	98.46	181.85	0.83	6.13	1.3e-02
dat_351	505	435	471	81.74	82.59	98.97	121.08	1.14	5.21	2.2e-02
**ldh_4**	**188305**	**365**	**453**	**100.00**	**100.00**	**100.00**	**100.00**	**418.70**	**187210.51**	**1.0e-26**
**ldh_20**	**1626**	**418**	**453**	**100.00**	**100.00**	**100.00**	**100.00**	**3.63**	**713.83**	**1.0e-26**
ldh_40	424	418	453	64.68	64.68	100.00	154.61	0.96	0.04	8.5e-01
ldh_58	383	418	453	52.76	54.08	97.55	184.90	0.91	1.56	2.1e-01
ldh_62	848	418	453	67.99	67.99	100.00	147.08	1.95	145.83	1.0e-26
ldh_75	340	418	453	50.11	50.33	99.56	198.68	0.82	8.11	4.4e-03
ldh_158	548	418	453	88.08	88.96	99.01	112.41	1.32	17.39	3.0e-05
ldh_183	374	418	453	76.60	77.70	98.58	128.69	0.92	2.49	1.1e-01
ldh_184	662	418	453	72.41	72.85	99.39	137.27	1.51	54.96	1.0e-12
ldh_200	1147	418	453	99.78	100.00	99.78	100.00	2.84	339.35	1.0e-26
ldh_202	529	418	453	72.19	72.85	99.09	137.27	1.22	12.92	3.3e-04
ldh_223	495	418	453	69.32	72.19	96.02	138.53	1.20	6.43	1.1e-02
ldh_258	1012	418	453	79.25	79.47	99.72	125.83	2.35	246.50	1.0e-26
ldh_274	367	415	450	62.44	62.44	100.00	160.14	0.94	3.02	8.2e-02
ldh_279	1067	418	453	91.39	91.83	99.52	108.89	2.44	283.41	1.0e-26
ldh_403	440	418	453	90.07	92.05	97.84	108.63	1.06	0.54	4.6e-01
ldh_414	474	418	453	73.95	74.17	99.70	134.82	1.06	3.47	6.3e-02
ldh_416	608	418	453	45.92	45.92	100.00	217.79	1.38	35.05	3.7e-09
ldh_429	447	418	453	60.71	60.93	99.64	164.13	1.07	0.95	3.3e-01
ldh_432	708	418	453	96.91	100.00	96.91	100.00	1.67	74.50	1.0e-17
ldh_446	1220	418	453	74.61	74.61	100.00	134.02	2.83	392.46	1.0e-26
ldh_511	491	418	453	85.21	85.43	99.74	117.05	1.11	5.80	1.6e-02
ldh_559	727	418	453	69.54	69.98	99.37	142.90	1.76	83.20	1.0e-19
ldh_624	490	418	453	45.47	45.47	100.00	219.90	1.08	5.65	1.7e-02
ldh_635	429	418	453	96.47	100.00	96.47	100.00	1.05	0.13	7.2e-01
ldh_642	451	418	453	61.81	62.25	99.29	160.64	1.09	1.22	2.7e-01
ldh_670	460	418	453	59.60	61.15	97.47	163.54	1.09	1.97	1.6e-01
ldh_687	792	418	453	70.64	71.08	99.38	140.68	1.82	115.39	1.0e-26
**lhkA_1**	**139227**	**402**	**480**	**100.00**	**100.00**	**100.00**	**100.00**	**291.03**	**138025.18**	**1.0e-26**
lhkA_15	636	443	480	58.12	59.17	98.24	169.01	1.47	34.41	5.0e-09
lhkA_16	1473	443	480	70.83	70.83	100.00	141.18	3.09	553.66	1.0e-26
lhkA_34	1824	442	480	65.00	65.42	99.36	152.87	4.05	841.39	1.0e-26
lhkA_40	546	443	480	46.88	47.29	99.12	211.45	1.19	10.66	1.1e-03
lhkA_48	588	443	480	83.12	84.17	98.76	118.81	1.27	20.30	6.6e-06
lhkA_78	500	443	480	95.21	95.83	99.35	104.35	1.10	3.41	6.5e-02
lhkA_140	574	443	480	71.46	71.88	99.42	139.13	1.25	16.79	4.2e-05
lhkA_167	376	443	480	63.33	63.75	99.35	156.86	0.85	5.54	1.9e-02
lhkA_186	709	443	480	51.46	52.08	98.80	192.00	1.59	61.29	1.0e-14
lhkA_187	908	443	480	99.79	100.00	99.79	100.00	2.04	159.89	1.0e-26
lhkA_211	503	443	480	61.46	61.67	99.66	162.16	1.08	3.76	5.2e-02
lhkA_213	772	443	480	61.04	61.25	99.66	163.27	1.72	88.94	1.0e-20
lhkA_221	1037	443	480	99.17	100.00	99.17	100.00	2.32	238.25	1.0e-26
lhkA_225	686	443	480	66.25	66.46	99.69	150.47	1.46	52.18	1.0e-12
lhkA_294	668	443	480	70.00	70.21	99.70	142.43	1.52	45.45	5.3e-10
lhkA_311	693	443	480	65.21	65.42	99.68	152.87	1.56	54.89	1.0e-12
lhkA_315	801	443	480	83.96	84.38	99.51	118.52	1.75	102.87	1.0e-23
lhkA_323	431	443	480	69.79	70.62	98.82	141.59	0.96	0.17	6.8e-01
lhkA_325	444	443	480	43.96	43.96	100.00	227.49	0.96	0.00	9.8e-01
lhkA_384	445	443	480	64.79	65.21	99.36	153.35	0.99	0.00	9.6e-01
lhkA_389	1024	445	483	98.96	99.17	99.79	100.84	2.23	227.33	1.0e-26
lhkA_403	1246	443	480	97.29	97.29	100.00	102.78	2.70	381.65	1.0e-26
lhkA_417	2167	442	480	11.46	12.50	91.67	800.00	4.95	1139.07	1.0e-26
lhkA_427	715	443	480	57.50	57.50	100.00	173.91	1.55	63.76	1.0e-14

Notes on naming conventions for metagenomic sequence files

This concerns metagenomic sequencing data deposited in NCBI BioProject PRJNA1178869.

Drain A in the publication corresponds to drain 8 in the sequencing files. Correspondingly, drain 9=B, 10=C, 11=D, 12=E, and 13=F.

Week 1 in the publication corresponds to week 18 in the sequencing files. Correspondingly, week 7=24, 8=25, and 9=26.

References

1. Fagerlund A, Wagner E, Møretrø T, Heir E, Moen B, Rychli K, Langsrud S. 2022. Pervasive *Listeria monocytogenes* is common in the Norwegian food system and is associated with increased prevalence of stress survival and resistance determinants. *Appl Environ Microbiol* 88:e0086122.
2. Ivanova M, Kragh ML, Szarvas J, Tosun ES, Holmud NF, *et al*. 2023. Large-scale phenotypic and genomic characterization of *Listeria monocytogenes* susceptibility to quaternary ammonium compounds. *bioRxiv* https://doi.org/10.1101/2023.09.07.556668.
